# Application of Multiplatform Mass Spectrometry to the Study of *Babesia divergens* Metabolism and the Pathogenesis of Human Babesiosis

**DOI:** 10.3390/ijms26167677

**Published:** 2025-08-08

**Authors:** Miguel Fernández-García, Luis Miguel Gonzalez, Elena Sevilla, Aitor Gil, Henrique Santos-Oliveira, Belen Revuelta, Coral Barbas, Mª Fernanda Rey-Stolle, Estrella Montero, Antonia García

**Affiliations:** 1 Centro de Metabolómica y Bioanálisis (CEMBIO), Facultad de Farmacia, Universidad San Pablo-CEU, CEU Universities, Urbanización Montepríncipe, 28660 Boadilla del Monte, Spain; miguel.fernandezgarcia@ceu.es (M.F.-G.); henrique.dosoliveira@ceu.es (H.S.-O.); cbarbas@ceu.es (C.B.); frstolle@ceu.es (M.F.R.-S.); 2 Departamento de Ciencias Médicas Básicas, Facultad de Medicina, Universidad San Pablo-CEU, CEU Universities, Urbanización Montepríncipe, 28660 Boadilla del Monte, Spain; 3 Parasitology Reference and Research Laboratory, Centro Nacional de Microbiología, Instituto de Salud Carlos III, Majadahonda, 28220 Madrid, Spain; lmgonzal@isciii.es (L.M.G.); esevilla@isciii.es (E.S.); aitor.gil@edu.uah.e (A.G.); belen.bnrd@gmail.com (B.R.)

**Keywords:** *Babesia divergens*, *Babesia divergens* metabolism, human babesiosis, multiplatform metabolomics

## Abstract

*Babesia divergens* is a tick-borne apicomplexan parasite that causes human babesiosis, a malaria-like disease. *B. divergens* metabolism remains poorly characterized. Here, we employed a multiplatform mass spectrometry-based metabolomics approach (using CE-TOF/MS, GC-QTOF/MS, LC-QTOF/MS, and LC-QqQ/MS) to profile intra- and extracellular metabolic changes in *B. divergens*-infected and uninfected red blood cells (RBCs) and their supernatants. Our results indicate alterations in the metabolome caused by *B. divergens* infection and proliferation within RBCs. These findings are consistent with the major metabolic dependencies of *B. divergens*, including extracellular glucose, glutamine, and arginine, accompanied by the accumulation of glycolytic and TCA cycle intermediates. We identified altered nucleotide metabolism, pentose phosphate pathway activity, and redox imbalance. Depletion of lysoglycerophospholipids, glucose, arginine, and glutamine, and accumulation of free heme and sphingolipids suggested pathogenic effects. Growth experiments indicate that glucose and glutamine, but not hypoxanthine, are required for parasite growth. We additionally discovered a phosphorylated HEPES derivative (PEPES) produced upon *B. divergens* infection of RBCs in vitro. Collectively, these findings and their global interpretation provide insights into *B. divergens* metabolism and metabolic dependencies and host–parasite metabolic interactions and outline potential directions for future studies on human babesiosis diagnosis, prognosis assessment, and treatment.

## 1. Introduction

Human babesiosis, caused by apicomplexan parasites of the genus *Babesia*, is a zoonotic emerging disease [1]. *Babesia* spp. are transmitted naturally by ticks. However, transmission by contaminated blood transfusion is possible [2]. *Babesia* parasites invade and infect red blood cells (RBCs), where they multiply, causing pathological symptoms. The severity of human babesiosis can vary, ranging from mild symptoms to life-threatening disease, depending on the specific *Babesia* species involved and the age and immune status of the infected individual [3]. Among these are *Babesia divergens*, which causes redwater fever in cattle [4] and babesiosis in humans [5]. *B. divergens* is transmitted by *Ixodes ricinus*, a common tick that is widely established in north-western Europe [5]. The suggested influence of climate change on the distribution of *I. ricinus* and the spread of tick-borne diseases, including babesiosis, in current and new areas is very interesting [6]. Cattle infected with *B. divergens* undergo high fever, listlessness, dehydration, and “pipestem” diarrhea. In the absence of treatment, the infection progresses to severe hemolytic anemia, alongside hypoxia, severe jaundice, and toxemic shock, and animals are recumbent or show excitability and aggressive behavior [4]. Clinical manifestations in humans infected with *B. divergens* are generally mild and nonspecific, including fever, chills, headache, and fatigue. Severe babesiosis results in anemia, jaundice, hemoglobinuria, splenomegaly, renal failure, and even death, particularly in immunocompromised patients [5].

*Babesia divergens* requires both sexual and asynchronous asexual life cycles to ensure transmission between the tick vector and the vertebrate host [7]. The infection begins when sporozoites are transmitted into the bloodstream of the vertebrate host through the saliva of an infected *I. ricinus* tick [7,8]. These sporozoites invade RBCs, where they develop into trophozoites and replicate asexually via merogony. This process gives rise to multiple daughter cells that differentiate into merozoites, the infective forms responsible for continued RBC invasion. Thus, the asexual life cycle is marked by the infectivity of the *B. divergens* free merozoite that efficiently enters RBCs. Once within the RBC, *B. divergens* builds a progeny of main and transitory intraerythrocytic parasites in a chronological order to generate sexual stage parasites and produce and release new merozoites capable of invading uninfected RBCs [9]. This ingenious strategy allows *B. divergens* to adapt to external conditions while establishing successful parasitism in the host bloodstream [10]. However, as part of its asexual life cycle, the parasite destroys RBCs, releasing intracellular contents into the bloodstream, thus leading to clinical manifestations of babesiosis. Moreover, a subset differentiates into gametocytes, which are non-dividing, sexually committed forms taken up by the tick during a blood meal on an infected host [7].

Within the midgut lumen of the ticks, gametocytes develop into gametes—also referred to as Strahlenkörper—which fuse to form diploid zygotes. These zygotes invade the intestinal epithelial cells, complete their development, and undergo meiosis to generate haploid primary kinetes. The primary kinetes disseminate via the hemolymph to various tissues, where they undergo further asexual multiplication to produce secondary kinetes [7]. In species belonging to the *Babesia* sensu stricto clade, such as *B. divergens*, primary kinetes can invade the ovaries of the adult female tick, enabling transovarial transmission to their offspring [7]. Secondary kinetes migrate to the salivary glands of the tick, where they differentiate into sporoblasts. These remain dormant through the tick’s molting stages, enabling transstadial transmission. Upon tick feeding, the sporoblasts are activated and give rise to new sporozoites, which are released into saliva and transmitted to the vertebrate host, thus completing the life cycle [7].

Current treatment options for human babesiosis are limited to a few antimicrobials and antimalarial agents. Mild cases are typically treated with a combination of atovaquone and azithromycin or, alternatively, clindamycin and quinine, which are also used in conjunction with complete RBC exchange transfusion in severe cases. These combination therapies were not developed based on the biological features of *Babesia* parasites. Instead, they were repurposed from drugs already known to be effective against other Apicomplexan pathogens. However, they are frequently associated with adverse effects, ranging from mild to severe, as well as rapid drug resistance. These challenges highlight the need for innovative therapeutic strategies specifically targeting the biology of *Babesia* [11].

The metabolism of piroplasmid apicomplexa, including *Babesia* spp., remains largely unexplored. Early biochemical studies in *Babesia rodhaini* revealed increased glucose consumption, lactate production, and oxygen uptake in parasitized RBCs [12]. Similar observations were documented in *Babesia bovis* [13] and *Babesia microti* [14]. Additional studies investigated the tricarboxylic acid (TCA) cycle and glycolytic enzyme activities in *B. microti* and *B. rodhaini* [15], characterized components of a functional TCA cycle in *Babesia gibsoni* [16], and identified an active purine salvage pathway in *B. bovis* [17] and *B. divergens* [18]. With the advent of high-throughput sequencing technologies, information on the global metabolic network of *Babesia* spp. metabolism has been described. For instance, genomic analysis of *B. microti* revealed a putative topology of the central carbon metabolism [19], while sequencing of the apicoplast genome in other *Babesia* species has revealed putative pathways of this organelle [20,21]. In contrast, little is known regarding *B. divergens* metabolism. Although its genome and transcriptome have been successfully sequenced and annotated [22,23], its metabolism remains poorly characterized from a metabolomic perspective. While metabolomics has been proven valuable in elucidating metabolic pathways and drug mechanisms in related apicomplexan parasites, such as *Plasmodium* spp. [24,25,26,27], the only up-to-date study of *B. divergens* [28] focused exclusively on the intracellular media of infected RBCs. The extracellular metabolic consequences of *B. divergens* parasitization, such as nutrient uptake and excretion of metabolic waste and metabolite secretion, remain largely unknown from a global point of view.

To characterize the metabolism of *B. divergens* and elucidate potential mechanisms underlying the pathophysiological effects of human babesiosis, three analytical platforms were used to perform a metabolic profiling with broad metabolite coverage of *B. divergens*-infected and uninfected RBCs (iRBCs and uRBCs, respectively) and their supernatants. These were capillary electrophoresis coupled to time-of-flight mass spectrometry (CE-TOF/MS), gas chromatography coupled to quadrupole-time-of-flight mass spectrometry (GC-QTOF/MS), and liquid chromatography coupled to quadrupole-time-of-flight mass spectrometry (LC-QTOF/MS) operating in positive (LC-ESI(+)-QTOF/MS) and negative (LC-ESI(-)-QTOF/MS) ionization modes. Additionally, these analyses were combined with a targeted analysis of polar compounds using liquid chromatography coupled to triple quadrupole mass spectrometry (LC-QqQ/MS), enabling the quantification of selected metabolites in iRBCs and uRBCs isolated from in vitro cultures. By integrating both semi-quantitative and quantitative metabolomics data from both intracellular and extracellular compartments with untargeted and targeted data from multiple analytical platforms and growth experiments, we aimed to obtain metabolic information in addition to that already reported using complementary targeted LC-MS methods [28]. This study provides new insights into the nutrient sources and their influence on parasite proliferation and is intended to generate hypotheses for further validation of metabolic pathway alterations and pathophysiological features associated with *B. divergens* infection.

## 2. Results

### 2.1. Metabolite Coverage and Univariate and Multivariate Analysis of Infected and Non-Infected RBCs and Supernatants

**Figure 1:**
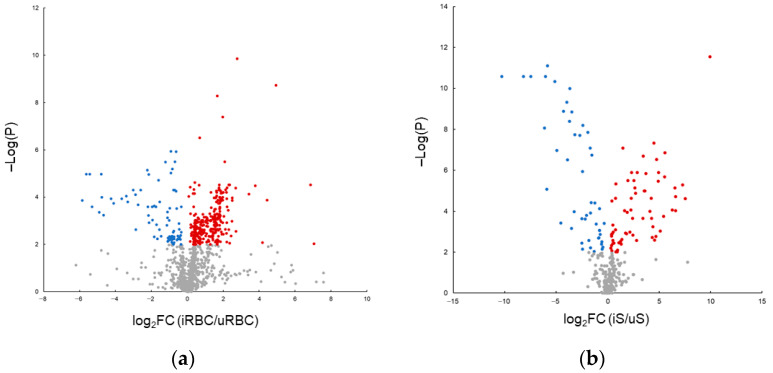
Volcano plots showing differences in abundance and statistical significance of annotated metabolites: (**a**) comparison between iRBCs and uRBCs; (**b**) comparison between iSs and uSs. Metabolites with increased relative abundance in iRBCs or iSs are shown in red; metabolites with decreased abundance are shown in blue; non-significant metabolites are shown in grey. An FDR cutoff of 0.01 was applied to highlight statistically significant differences.

**Figure 2:**
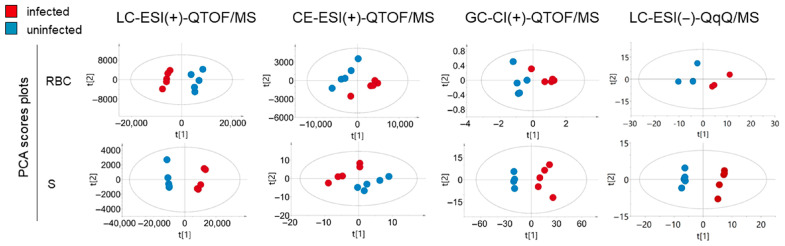
PCA score plots of multivariate models generated for iRBCs vs. uRBCs and iSs vs. uSs comparisons across all applied metabolomic platforms. iRBC and iS samples are shown in red. uRBC and uS samples are shown in blue.

Using the described multiplatform approach, 1203 molecular features were detected in RBC extracts. Among these, compatible annotations were found for 268 metabolites (Table S1). Univariate statistical analysis revealed a total of 411 significant molecular features distinguishing iRBC and uRBC samples (Figure 1a). Exploratory principal component analysis (PCA) score plots showed clear group separation and clustering between iRBC and uRBC samples (Figure 2).

Chemical and metabolite set over-representation analysis performed in the annotated metabolites determined that samples obtained from iRBCs, uRBCs, and supernatants were highly similar in terms of qualitative composition. Overall, the annotated metabolite dataset was enriched in distinct chemical subclasses, including amino acids, small organic acids, monosaccharide derivatives, purine and pyrimidine-related compounds, and, to a minor extent, in subclasses, including sphingolipids, pyridine-related compounds, and phosphate esters, achieving a varied coverage of the chemical space of the metabolome (Figure S1A,B).

MS data obtained from iS and uS extracts were screened for molecular features present in the iRBC and uRBC samples. A total of 284 molecular features were detected in supernatant extracts, 188 of which were annotated metabolites. Univariate analysis revealed 99 significant molecular features differentiating iS and uS samples (Figure 1b). Multivariate analyses of iS and uS samples showed similar trends to those observed in iRBC and uRBC samples, with clear clustering and distinction between iS and uS samples in PCA models (Figure 2, Table 1).

This metabolome coverage enabled the semiquantitative evaluation of pathways represented by the dataset, including those belonging to the metabolism of amino acids (arginine biosynthesis, aminoacyl-tRNA biosynthesis, alanine, aspartate and glutamate metabolism, phenylalanine, tyrosine and tryptophan metabolism, glutathione metabolism, arginine and proline metabolism, valine, and isoleucine and leucine metabolism), nucleotides (pyrimidine and purine metabolism), carbohydrates (galactose metabolism, pentose phosphate pathway, and aminosugar and sugar nucleotide metabolism), lipids (glycerophospholipid metabolism, biosynthesis of unsaturated fatty acids, and sphingolipid metabolism), vitamins and cofactors (nicotinate and nicotinamide metabolism and vitamin B6 metabolism) and the TCA cycle (Figure S2A,B).

It is noteworthy that six metabolites were only detected in iRBCs (2-deoxyadenosine-5-monophosphate, thymidine, thymine, oxalacetate, gamma-glutamylcysteine, and trehalose), and three metabolites (lysophosphatidylcholine (LPC) 18:1, LPC (19:0), and a hexose phosphate) were only detected in uRBCs (Table S1). Similarly, a subset of metabolites was only detected in iS samples, including two purine-related metabolites (2-deoxyadenosine-5′-monophosphate and ADP), four pyrimidine-related metabolites (2-deoxycytidine, 2-deoxycytidine-5′-diphosphate, thymidine, and a UDP-hexose), 2-hydroxyglutarate, nicotinic acid mononucleotide, fructose-6-phosphate, dihydrosphingosine, and free heme, while a hexosylhexose and thymidine-5′-diphosphate were only detected in uSs. Collectively, this data indicates very high or qualitative differences in the profile of infected RBCs and their extracellular media (Table S1).

### 2.2. Representation and Significance of Altered Metabolic Pathways in Infected RBCs and Supernatants

**Figure 3:**
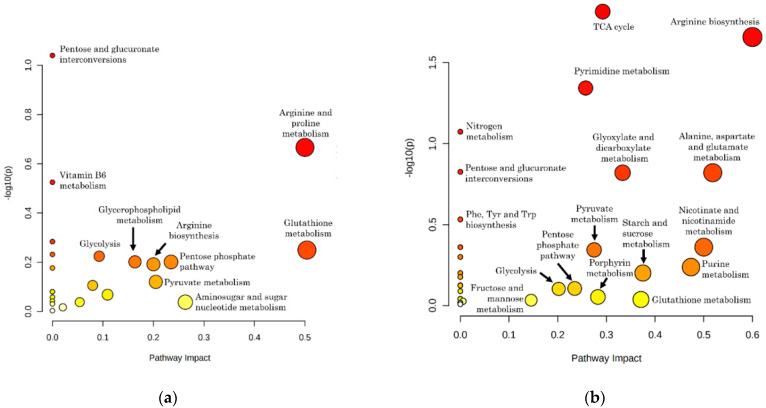
Pathway analysis of significantly altered metabolic pathways in *B. divergens*-infected vs. uninfected RBC cultures. (**a**) Pathway analysis results for iRBC vs. uRBC comparison; (**b**) pathway analysis results for iS vs. uS comparison.

Initial insights into metabolic pathway alterations induced by *B. divergens* were obtained through independent pathway analyses performed on the subsets of statistically significant metabolites determined in iRBC vs. uRBC and iS vs. uS comparisons (Figure 3). Several pathways showed low *p*-values and high pathway impact scores in both comparisons (Figure 3). Notably, the comparison between iS and uS revealed a more extensively altered metabolic profile. Among the most affected pathways were arginine biosynthesis, the TCA cycle, pyrimidine metabolism, glyoxylate and dicarboxylate metabolism, alanine, aspartate and glutamate metabolism, the pentose phosphate pathway, nitrogen metabolism, nicotinate and nicotinamide metabolism, purine metabolism, pyruvate metabolism, interconversions between pentoses and glucuronates, starch and sucrose metabolism, porphyrin metabolism, phenylalanine, tyrosine and tryptophan biosynthesis, fructose and mannose metabolism, and glycolysis (Figure 3). In contrast, the iRBC vs. uRBC comparison highlighted alterations in glycolysis, glutathione metabolism, and the pentose phosphate pathway, all of which exhibited lower *p*-values relative to the iS vs. uS comparison. Interestingly, arginine and proline metabolism, glycerophospholipid metabolism, aminosugar and sugar nucleotide metabolism, and vitamin B6 metabolism were specifically enriched in iRBCs vs. uRBCs and were not significantly altered in the iS vs. uS analysis (Figure 3), indicating a wide variety of affected metabolic pathways upon *B. divergens* infection, including ones not reflected in the extracellular media.

**Figure 4:**
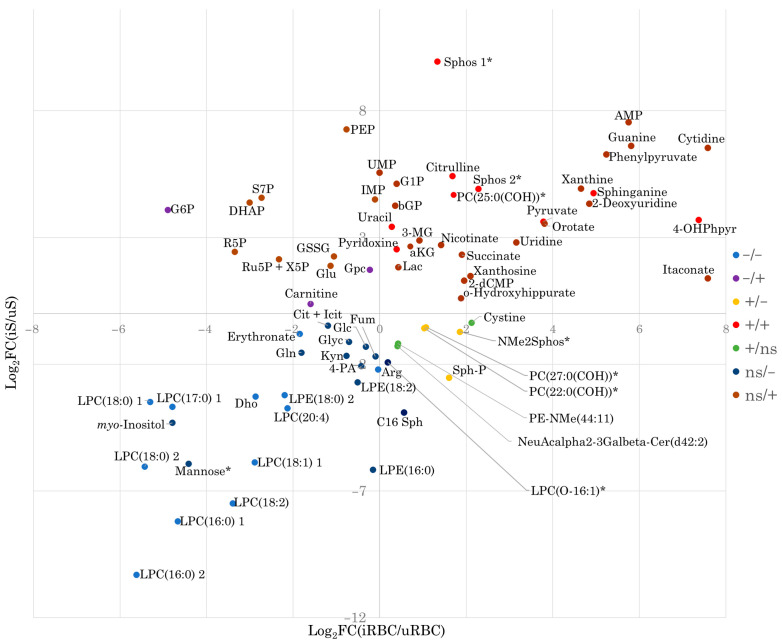
Changes in relative abundances of metabolite groups detected in iRBCs, uRBC, iSs, and uSs, classified based on statistical significance and the sign of log2FC. Metabolites were grouped according to the trend in mean values in iRBCs and iSs, denoted as [iRBC trend in means]/[iS trend in means]. In this context, “+” indicates a statistically significant increase in relative abundance; “-“ indicates a statistically significant decrease; “ns” denotes non-significant changes. The most probable annotation is indicated for metabolites marked with an asterisk, “*”. Only metabolites with log_2_FC ≥ 1 in at least one comparison were included to facilitate data representation. Metabolites are abbreviated as follows: 2-dCMP, 2-deoxycytidine-5′-monophosphate; 3-MG, 3-methylglutarate; 4-PA, 4-pyridoxate; 4-OHPhPyr, 4-hydroxyphenylpyruvate; AMP, adenosine-5′-monophosphate; aKG, alpha-ketoglutarate; Arg, arginine; bGP, beta-glycerophosphate; C16 Sph, C16 Sphinganine; Cer, ceramide Cit, citrate; DHAP, dihydroxyacetone phosphate; Dho, Dihydroorotate; Fum, fumarate; G1P, glycerol-1-phosphate; G6P, glucose-6-phosphate; Glc, glucose; Gln, Glutamine; Glu, glutamate; Glyc, glycerate; Gpc, glycerophosphocholine; GSSG, oxidized glutathione; Icit, isocitrate; IMP, inosine-5′-monophosphate; Kyn, kynurenine; Lac, lactate; LPC, lysophosphatidylcholine; LPE, lysophosphatidylethanolamine; MTA, 5′-methylthioadenosine; NAcNeu, N-acetylneuraminate; NMe2Sphos, N,N-dimethylsphingosine; PC, phosphatidylcholine; PE-NMe, N-methylphosphatidylethanolamine; PEP, phosphoenolpyruvate; R5P, ribose-5-phosphate; Ru5P, ribulose-5-phosphate; S7P, sedoheptulose-7-phosphate; SAH, S-adenosylhomocysteine; Sphos, sphingosine; Sph-P, sphinganine phosphate; TC, taurocholate; UMP, uridine-5′-monophosphate; X5P, xylulose-5-phosphate.

To provide further insights into the compartment-specific metabolic changes associated with *B. divergens* RBC infection, we performed pathway analysis and chemical class over-representation analysis of metabolite subsets, classified according to both their statistical significance and directionality of change in the comparisons of iRBCs vs. uRBCs and iS vs. uS (Table 1 and Table S1, Figure 4). Grouping metabolites’ chemical structure and pathway involvement enabled the identification of shared and divergent patterns across compartments, offering a contextualized view of the metabolic alterations induced by infection.

Notably, sphingolipids (sphinganine and sphingosine), citrulline, uracil, pyridoxine, pyruvate, and 4-hydroxyphenylpyruvate were contained in the subset of metabolites elevated in both iRBCs and iS samples (Figure 4). Most of the metabolites significantly increased in iS did not reach statistical significance in the iRBC vs. uRBC comparison. This subset exhibited marked enrichment in, among others, pyrimidine and purine-related compounds (uracil, 2-deoxyuridine, thymidine, uridine-5′-monophosphate, 2-deoxycytidine-5′-monophosphate, adenosine-5′-monophosphate, adenosine-5′-diphosphate, 2-deoxyadenosine-5′-monophosphate, inosine-5′-monophosphate, and guanine), as well as in TCA cycle intermediates (e.g., α-ketoglutarate and succinate), short-chain acids (pyruvate and phosphoenolpyruvate), phosphate esters (glycerol-1-phosphate and β-glycerophosphate), and phenylpyruvate derivatives (phenylpyruvate) (Table 1, Figure 4).

Cystine significantly increased in iRBCs but not in iS (Table 1, Figure 4). Several pentose phosphate pathway metabolites (sedoheptulose-7-phosphate, ribose-5-phosphate, xylulose-5-phosphate + ribulose-5-phosphate, glucose-6-phosphate) were increased in iS but showed non-significant changes in iRBCs, except for glucose-6-phosphate, which was significantly decreased in RBCs. All measured pentose phosphate compounds showed negative log_2_ fold-change (log_2_FC) values in iRBCs (Table 1, Figure 4). A similar trend was observed for glycerophosphocholine and carnitine.

Several phospholipids and sphingolipids showed an opposite trend to the above-mentioned compounds, being significantly increased in iRBCs and decreased in iS (Figure 4). In addition, among metabolites consistently depleted in both compartments, several lysophosphatidylcholines (LPCs) and dihydroorotate were markedly reduced (Table 1, Figure 4). LPC depletion coincided with significantly decreased levels of glucose, glutamine, arginine, glycerol, kynurenine, citrate (citrate + isocitrate), and fumarate (Table 1, Figure 4). Despite these reductions in iS, their levels did not differ significantly between iRBCs and uRBCs (Figure 4).

Lastly, the evaluation of the compounds uniquely detected in iRBCs and uRBCs but absent in iS and uS revealed a limited number of metabolites, characterized by significantly decreased relative abundances of spermidine, isobutirylcarnitine, and butyrylcarnitine, in conjunction with significantly increased levels of phosphocholine and S-glutathionylcysteine (Table S1).

### 2.3. Absolute and Relative Concentrations of Metabolites in the B. divergens Intraerythrocytic Parasite–RBC System

**Figure 5:**
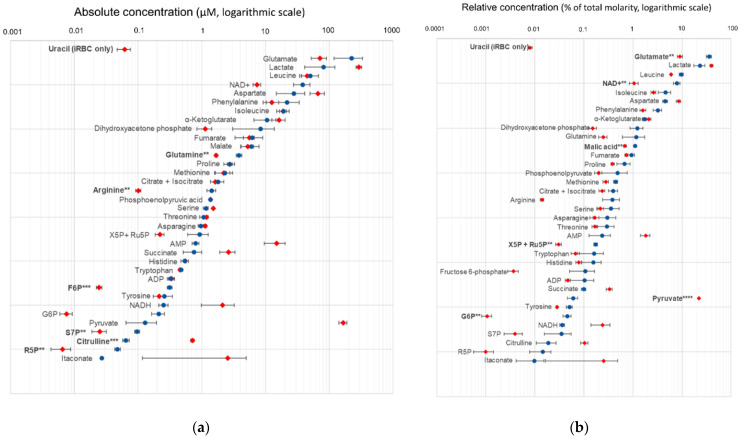
Differential analysis of metabolite concentrations between iRBC and uRBC extracts, confirmed by authentic standards. (**a**) Absolute concentrations; (**b**) relative concentrations. iRBC extract mean values are shown in red; uRBC extract mean values are shown in blue. Error bars correspond to standard error of measured metabolites. Only compounds above limit of quantification (signal-to-noise ratio > 10) are shown. DHAP, dihydroxyacetone phosphate; F6P, fructose-6-phosphate; R5P, ribose-5-phosphate; Ru5P, ribulose-5-phosphate; S7P, sedoheptulose-7-phosphate; X5P, xylulose-5-phosphate; **: *p* < 0.01; ***: *p* < 0.001; ****: *p* < 0.0001.

To further investigate the intracellular metabolome of *B. divergens* and its relationship with the host RBC, we determined the absolute and relative concentrations (i.e., the percentage of contribution to total molar content) of 36 metabolites previously confirmed by authentic standards in iRBCs and uRBCs using a targeted LC-QqQ/MS analysis. The metabolite panel included selected amino acids, pentose phosphate intermediates, TCA cycle metabolites, glycolytic intermediates, uracil, ADP, ATP, NAD+, and NADH (Figure 5a,b and Table S2). Measured concentrations ranged from high micromolar (~0.5 mM) to nanomolar (~6 nM) (Figure 5a, Table S2).

Lactate, glutamate, and aspartate accounted for more than 50% of the total quantified intracellular metabolite pool across both sample types (Figure 5b and Figure S3). A statistical comparison of absolute concentrations between iRBCs and uRBCs identified two significantly increased metabolites (pyruvate and citrulline) and five significantly decreased compounds (glutamine, arginine, fructose-6-phosphate, sedoheptulose-7-phosphate, and ribose-5-phosphate) (Figure 5a). Analysis of relative concentrations revealed six additional statistically significant metabolites with decreased levels in iRBCs: malate, glutamate, NAD+, glucose-6-phosphate, and a combined pool of xylulose-5-phosphate + ribulose-5-phosphate. These findings also confirm a significant relative increase in pyruvate levels (Figure 5b). Additionally, several metabolites exhibited notable differences in metabolite concentrations; however, these did not reach statistical significance. These included lactate, aspartate, AMP, succinate, NADH, and itaconate. Decreased levels of dihydroxyacetone phosphate were observed (Figure 5a,b). Notably, uracil was exclusively detected in iRBC samples.

### 2.4. Structural Elucidation of Phosphorylated HEPES, a Novel Metabolite Uniquely Detected in iRBCs

**Figure 6:**
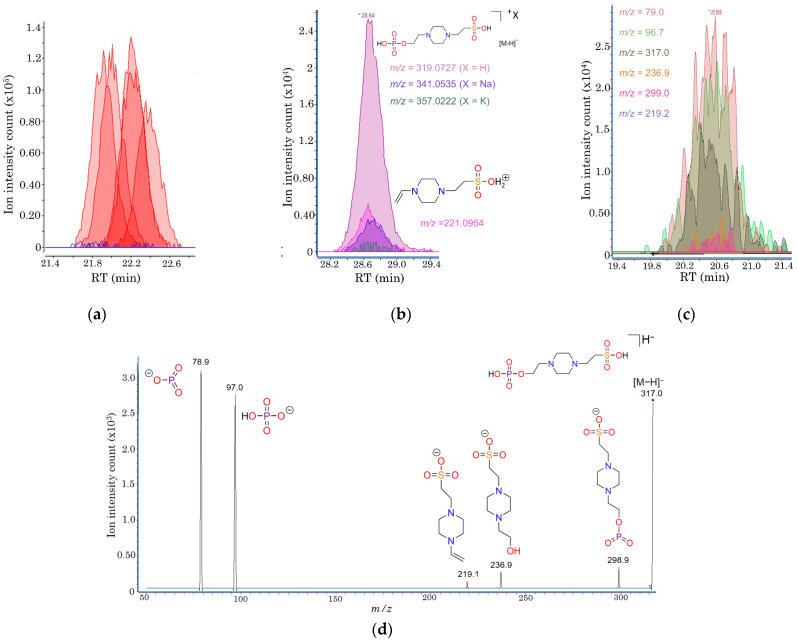
Structural elucidation of 2-(4-(2-(phosphonooxy)ethyl)piperazinyl)-ethanesulfonic acid (PEPES). (**a**) An elecropherogram showing migration time and signal abundances of PEPES in CE-ESI(+)-TOF/MS-analyzed samples; (**b**) detected adducts and in-source fragments of in an iRBC pooled extract analyzed by CE-ESI(+)-TOF/MS at 200 V with the associated candidate structures for the observed adducts and fragments; (**c**) retention time, abundance, and peak shape of PEPES-attributed *m*/*z* obtained from an LC-ESI(-)-QqQ/MS/MS-analyzed RBC extract; (**d**) MS/MS spectra corresponding to the PEPES in an LC-ESI(-)-QqQ/MS/MS-analyzed RBC extract, with candidate structures for the observed fragments. PEPES *m*/*z* traces corresponding to the [M+H]^+^ adduct shown in (**a**) were depicted in red for iRBC extracts and in blue for uRBC extracts.

Using the multiplatform mass-spectrometry pipeline described above, more than 1200 molecular features were detected in the untargeted analysis of iRBCs and uRBCs. From these, a prominent feature present in iRBCs, showing only spurious signals in uRBCs, was detected by CE-ESI(+)-TOF/MS (Figure 6a). The migration time of this molecular feature was slightly higher than the EOF (20.4 min), suggesting it could be a negatively charged compound at the acidic buffer pH (pH ≈ 3). Given the importance of this alteration and the inability to match with database annotations, further structural elucidation was pursued, as *B. divergens*-specific compounds not produced by the host may play key roles in parasite biology or pathogenesis. For this purpose, CE-ESI(+)-QTOF/MS data were evaluated, and complementary targeted LC-QqQ/MS analyses were performed (Figure 6; File S1). This approach culminated in the identification of 2-(4-(2-(phosphonooxy)ethyl)piperazinyl)-ethanesulfonic acid (PEPES), a phosphorylation of 4-(2-hydroxyethyl)-1-piperazineethanesulfonic acid, a buffering agent added to the cell cultures (HEPES, Figure 6b). This result indicates that HEPES is selectively phosphorylated in *B. divergens* RBC cultures (Figure 6c,d).

### 2.5. Functional Validation of Selected Extracellular Nutrients

**Figure 7:**
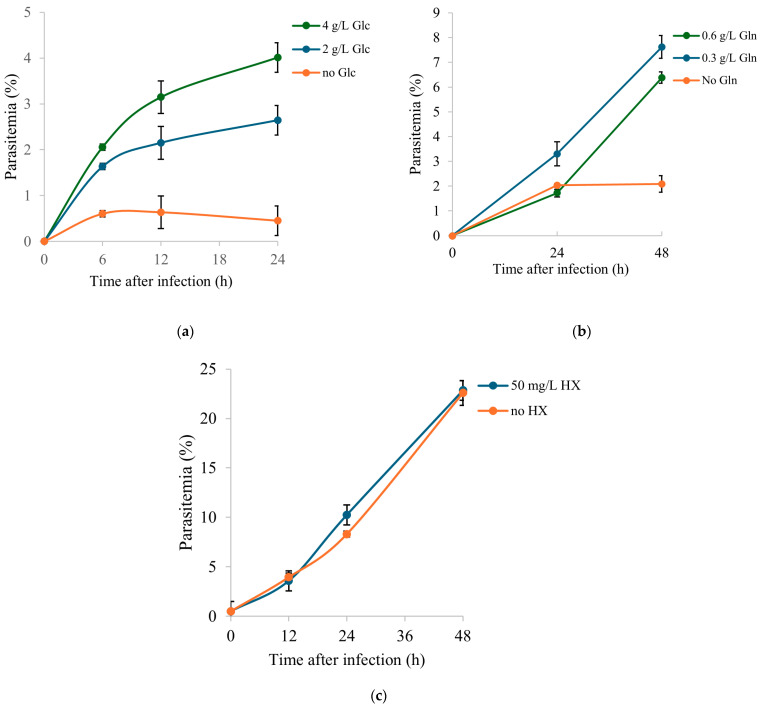
Growth kinetics of B. divergens under different nutrient supplementation conditions: (**a**) glucose; (**b**) glutamine; (**c**) hypoxanthine. Error bars represent the standard error of the mean (SEM) for three independent biological replicates (*n* = 3).

To investigate the biological relevance of selected extracellular nutrients identified as significantly consumed from the culture medium, *B. divergens* growth assays were performed under differential supplementation conditions (Figure 7 and Tables S3 and S4). The metabolites assessed included glucose, glutamine, and hypoxanthine.

The effect of extracellular glucose on parasite growth was under three glucose concentrations: an unsupplemented medium, mid glucose supplementation (2 g/L), and high glucose supplementation (4 g/L) (Figure 7a; Tables S3 and S4). Parasitemia was measured at 6 h, 12 h, and 24 h post-infection. At 6 h, only high glucose supported significantly higher parasitemia compared with the unsupplemented medium, while no differences were observed between mid-glucose supplementation and the other groups. At 12 h, both supplemented conditions showed significantly higher parasitemia than the unsupplemented medium, although no difference was detected between mid and high glucose supplementation. By 24 h, all concentrations differed significantly, with high glucose supporting the highest parasitemia, followed by mid glucose supplementation, and unsupplemented medium showing the lowest values. Parasitemia increased significantly between 6 h and 24 h under high glucose supplementation compared to medium glucose supplementation, the latter of which exhibited the expected trend of parasite growth (with parasitemia doubling approximately every 18 h). In contrast, no discernible parasite proliferation was observed in the glucose-deprived medium. These results demonstrate that extracellular glucose is required for parasite growth and that proliferation is strongly dependent on glucose concentration.

The impact of extracellular glutamine availability was assessed under three conditions: an unsupplemented medium, mid-level supplementation (0.3 g/L), and high-level supplementation (0.6 g/L). Parasitemia was monitored at 24 h and 48 h post-infection. A significant increase in parasitemia was detected between 24 h and 48 h in both supplemented conditions, whereas no significant change over time was observed in the absence of glutamine (Figure 7b; Tables S3 and S4). At 48 h, parasitemia under glutamine supplementation (both 0.3 g/L and 0.6 g/L) was significantly higher compared to the unsupplemented condition, while differences between mid- and high-level supplementation were not significant at either time point. These data indicate that extracellular glutamine is required to support parasite growth.

The role of extracellular hypoxanthine was investigated by culturing parasites in an unsupplemented medium and a medium supplemented with hypoxanthine (50 mg/L), with growth monitored over four time points (0 h, 24 h, 48 h, and 72 h post-infection). Parasitemia increased progressively in both hypoxanthine-supplemented and non-supplemented cultures, with significant differences between consecutive time points (Figure 7c; Tables S3 and S4). Two independent experiments with reduced monitoring intervals (0 h, 24 h, and 48 h) reproduced the absence of significant differences between conditions (Tables S3 and S4). These findings indicate that hypoxanthine supplementation does not confer a growth advantage under the tested conditions in vitro.

## 3. Discussion

### 3.1. Discriminatory Profile and Potential Biomarker Utility of B. divergens-Induced Metabolic Alterations

In this study, the application of a multiplatform MS metabolomic approach reveals a notable number of significant metabolites and a highly discriminant profile found between iRBCs and uRBCs, as well as between iS and uS. These results provide preliminary evidence of intracellular and extracellular metabolic alterations during *B. divergens* infection and development within RBCs in vitro. Notably, PCA group separation and univariate statistical analysis indicate that, based on the measured metabolite abundances, iRBC and iS samples are strongly discriminated from uRBC and uS samples, respectively. Collectively, our results point to highly discriminant metabolites present in the supernatant or inside RBCs, whose potential as biomarkers for diagnosis, prognosis, and treatment efficacy assessment could be further evaluated in vitro and in vivo in independent studies.

Given the in vitro nature of our study, several aspects of experimental design must be considered when further evaluating the performance of identified potential biomarkers. Although some compounds were detected exclusively in the iRBC or iS samples, their abundances were relatively low. Moreover, the evaluated in vitro model showed high parasitemia levels (~40%), whereas most human babesiosis patients typically present with lower parasitemia rates [29]. As a consequence, a pre-concentration step may be necessary to monitor these compounds induced by *B. divergens* infection in in vivo studies. Secondly, certain metabolites (e.g., arginine and lysophosphatidylcholines) are also altered in *P. falciparum* infections, suggesting that the metabolic changes associated with *B. divergens* infection may not be sufficiently selective for the discrimination of *Babesia* spp. from other than *B. divergens*- or *Plasmodium falciparum*-infected patients.

Despite the above-stated limitations, the subset of altered metabolites found in our study may hold particular value for monitoring the progression of piroplasmid or plasmodial infections, complementary to progression assessment by level of parasitemia and other clinical and biochemical biomarkers currently used in clinical practice. In this context, our study provides a foundation for future validation studies aimed at identifying metabolite biomarkers in blood, serum, or plasma that reflect disease dynamics in human babesiosis caused by *B. divergens*.

### 3.2. Putative Metabolic Pathway Structure and Potential Carbon and Nitrogen Sources of B. divergens

**Figure 8:**
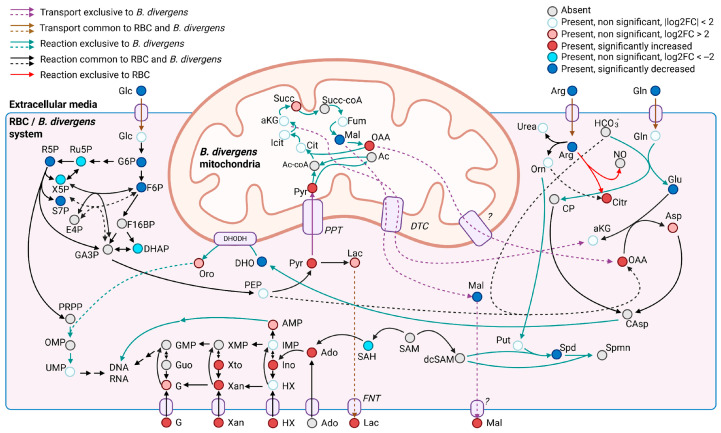
An integrated metabolic map of *B. divergens* in infected red blood cells (iRBCs). This schematic illustrates key metabolic pathways active in the system formed by *B. divergens* and the infected RBCs, including glycolysis, the pentose phosphate pathway, purine salvage and de novo pyrimidine biosynthesis, the tricarboxylic acid (TCA) cycle, polyamine biosynthesis, reactions of the urea cycle, and amino acid metabolism. Reactions and transports are represented by arrows, while metabolites are represented by circles. Transports exclusive to *B. divergens* are represented in purple; transports common to RBC and *B. divergens* are represented in brown; reactions exclusive to RBC are represented in red; reactions exclusive to *B. divergens* are represented in cyan; reactions common to RBC and *B. divergens* are represented in black. Non-detected metabolites are shown in grey; detected, non-significant metabolites with |log2FC| < 2 are shown in white; detected, non-significant metabolites with log2FC > 2 are shown in light red; detected, significantly increased metabolites are shown in dark red; detected, non-significant metabolites with log2FC < −2 are shown in light blue; detected, significantly decreased metabolites are shown in dark blue. Ado: adenosine; Ac: acetate; Ac-coA: acetyl-CoA; aKG: alpha-ketoglutarate; AMP: adenosine monophosphate; Arg: arginine; Asp: aspartate; AS: argininosuccinate; CAsp: carbamoyl-aspartate; Cit: citrate; Citr: citrulline; CP: carbamoyl phosphate; dcSAM: decarboxylated S-adenosylmethionine; DHAP: dihydroxyacetone phosphate; DHODH: dihydroorotate dehydrogenase; DHO: dihydroorotate; DNA: deoxyribonucleic acid; DTC: dicarboxylate transporter; E4P: erythrose-4-phosphate; F16BP: fructose-1,6-bisphosphate; F6P: fructose-6-phosphate; FNT: formate–nitrite transporter; Fum: fumarate; G: guanine; GA3P: glyceraldehyde-3-phosphate; G6P: glucose-6-phosphate; Glc: glucose; Gln: glutamine; GMP: guanosine monophosphate; Guo: guanosine; HCO3-: bicarbonate; HX: hypoxanthine; Icit: isocitrate; IMP: inosine monophosphate; Ino: inosine; Lac: lactate; Mal: malate; OAA: oxaloacetate; OMP: orotidine monophosphate; Orn: ornithine; Oro: orotate; PEP: phosphoenolpyruvate; PPT: phosphate transporter; PRPP: phosphoribosyl pyrophosphate; Put: putrescine; Pyr: pyruvate; R5P: ribose-5-phosphate; RNA: ribonucleic acid; Ru5P: ribulose-5-phosphate; S7P: sedoheptulose-7-phosphate; SAH: S-adenosylhomocysteine; SAM: S-adenosylmethionine; Spd: spermidine; Spmn: spermine; Succ: succinate; Succ-coA: succinyl-CoA; UMP: uridine monophosphate; X5P: xylulose-5-phosphate; XMP: xanthosine monophosphate; Xan: xanthine; Xto: xanthosine. Significance and fold-changes of LC-QqQ/MS quantified data were prioritized over non-quantified data reported in the manuscript.

*B. divergens* has a relatively small genome, and it has been suggested that the parasite is auxotrophic for several key metabolites, largely depending on host metabolite pools [28]. Initially, we focused on metabolites that could serve as carbon sources feeding into central carbon metabolism. We hypothesized that carbon substrates for *B. divergens* would be significantly depleted from the RBC content and/or the extracellular medium, if this RBC content was not sufficient for parasite activity and growth, and *B. divergens* had efficient uptake mechanisms. Glucose and glutamine are carbon sources in *Plasmodium* spp. and other *Babesia* species [12,13,19]. In our data, glucose levels decreased in the supernatants (Figure 4, Table S1), accompanied by increases in pyruvate and lactate levels (Figure 4, Figure 5 and Figure S3, Table S1) in both iRBCs and iSs. Together with these metabolic changes, growth assays demonstrated that extracellular glucose is required for parasite proliferation, with higher concentrations supporting significantly greater parasitemia at later time points (Figure 7a; Tables S3 and S4). As described for other *Babesia* species [12,13] and in line with the accumulation of pyruvate reported for iRBCs [28], these findings strongly suggest that *B. divergens* energy metabolism relies on glucose utilization through glycolysis as a major carbon source (Figure 8). However, as RBCs lack of a TCA cycle, the increased abundances of TCA cycle intermediates in iSs (Figure 3, Table 1), together with detectable levels of the unstable metabolite oxaloacetate in iRBCs and its absence in uRBCs (Table S2), suggests the presence of an operational TCA cycle in *B. divergens* (Figure 8), in accordance with previous evidence [28].

*Babesia* parasites efficiently replicate within iRBCs, which are devoid of nuclei and, therefore, lack significant quantities of nucleic acid intermediates other than ATP-related compounds. Consequently, *B. divergens* must synthesize pyrimidine nucleotides de novo to support DNA and RNA biosynthesis. While reliance on a purine salvage pathway for purine nucleotide production has been confirmed [18], a de novo pyrimidine biosynthesis pathway—initiated by the conversion of glutamine to glutamate—is present in *Babesia* species [30,31]. This pathway was underscored by PA in our study (Figure 3), and several purine- and pyrimidine-related compounds were significantly altered in iRBCs and iS (Figure 4, Table S1), in agreement with previously reported increased nucleotide-related compounds in *B. divergens*-infected RBCs [28]. Glutamine, beyond its involvement in central carbon metabolism, serves as a crucial carbon and nitrogen donor in orotate-dependent de novo pyrimidine biosynthesis [31]. Growth assays reinforce this interpretation, as supplementation with glutamine was required for parasite growth compared to the unsupplemented medium, although the effect was less pronounced than that observed for glucose (Figure 7b; Tables S3 and S4). In addition, alterations in alanine, aspartate, and glutamate metabolism found in PA, decreased absolute concentrations of glutamine, decreased relative concentrations of glutamate found in iRBCs (Figure 5 and Figure S3, Table S2), as well as decreased glutamine levels found in iSs (Figure 4, Table S1) indicate that both intracellular and extracellular glutamine and glutamate are consumed by *B. divergens*. Together with these observations, increases in α-ketoglutarate (in iSs) and glutamate (in both iSs and iRBCs) suggest that glutamine consumption may flux into the TCA cycle through a transaminase system. Collectively, these findings indicate that glutamine is required for *B. divergens* growth, primarily for its essential role as a nitrogen donor for de novo pyrimidine biosynthesis, while a secondary role as an alternative carbon source cannot be excluded (Figure 8). Within this context, inhibitors of dihydroorotate dehydrogenase, a key enzyme in de novo pyrimidine biosynthesis, have shown antiparasitic activity in *B. bovis* and may similarly affect *B. divergens* [32].

Additionally, it is well established that the growth media of *B. divergens* and other *Babesia* species are supplemented with hypoxanthine [33]. Hypoxanthine is taken up by the parasite, participating in purine nucleotide metabolic pathways and ultimately contributing to DNA and RNA biosynthesis [34]. In line with this, the observed concentrations of inosine and AMP in iRBCs (Figure 4, Table S1) may reflect increased utilization of purine metabolites during infection. However, growth assays showed no significant differences between hypoxanthine-supplemented and unsupplemented conditions within the tested timeframe (Figure 7c; Tables S3 and S4), indicating that hypoxanthine supplementation does not confer a growth advantage and suggesting a low dependency on extracellular hypoxanthine under these experimental conditions. This finding indicates that *B. divergens* may not rely heavily on exogenous hypoxanthine for proliferation due to the plasticity of the purine nucleotide salvage pathway (Figure 8), and supplementation might not be strictly necessary, although studies over longer incubation periods are warranted to confirm this. We hypothesize that this low dependency is presumably due to the sufficient purine nucleotide pool already present inside host RBCs, which could support parasite replication without additional supplementation.

Aside from the purine and pyrimidine-related DNA and RNA bases, nucleotides possess a sugar moiety that is generated from phosphoribosy lpyrophosphate (PRPP), a compound that originates in the pentose phosphate pathway. While both *B. divergens* and uninfected RBCs harbor this metabolic pathway, the significant enrichment of pentose phosphate pathway intermediates in both iRBCs and iSs (Figure 4, Table 1) suggests that *B. divergens* infection of RBCs leads to a notable alteration of this pathway. As such, glucose metabolism via the pentose phosphate pathway likely contributes not only to the generation of reducing power but also to the provision of sugar backbones essential for nucleotide biosynthesis in the system co-formed by *B. divergens* and the host RBC (Figure 8).

Arginine metabolism also emerged as a critical aspect of *B. divergens* metabolism. Marked depletion of arginine in iRBCs and iSs (Figure 4 and Figure 5, Table S1), suggests substantial uptake and further metabolism of arginine by the *B. divergens*–RBC system. Arginine has been described to be effectively captured from the extracellular media by *P. falciparum* via a high-affinity cationic transporter [35]. Once inside *P. falciparum*, arginine is fluxed through arginase and ornithine decarboxylase to form polyamines, a series of dibasic compounds necessary for DNA stability and replication [24,35]. Similarly, we hypothesize that polyamine formation might be a primary role of arginine acquisition by *B. divergens*, as the relevance of this compound has also been documented in distinct protozoa [36]. However, host-derived mechanisms underlying arginine depletion in iS aside from *B. divergens* consumption must be considered [37], such as the induction of host nitric oxide synthases upon infection [38]. In this context, significant increases in the levels of citrulline were also observed in both iRBCs and iSs (Figure 4 and Figure 5, Table S1), suggesting that arginine is not only converted to ornithine, undergoing subsequent polyamine biosynthesis, but also to a considerable amount of citrulline in the *B. divergens*–RBC system. Given the absence of a complete urea cycle in *Plasmodium* spp. [35] and the presence of nitric oxide synthase in RBCs, our results suggest that the high conversion rates of arginine to citrulline may not be entirely mediated by *Babesia* spp. Contrarily, a competition between the host RBC nitric oxide synthase converting arginine to citrulline and releasing toxic NO and *B. divergens* and host RBC arginase activity may be established upon *B. divergens* infection of RBCs. Given the depletion of arginine occurring in both iRBCs and iS, the apparent absence of a robust network arising from a full urea cycle and the intriguing increases in citrulline, we hypothesize that arginine and polyamine metabolism, as well as arginine transporters, may be of key relevance for *B. divergens* growth, whose role as potential targets for inhibitors could be evaluated in further studies

Having discussed the role of polar metabolites as nutrient sources, the potential of lipids as nutrient sources was evaluated. Specifically, dramatic decreases were observed in the relative abundance of lysophosphatidylcholines (LPCs) occurring in iRBCs and iS (Figure 4, Table S1), in line with previously reported evidence for *B. divergens*-infected RBCs [28]. Remarkably, the magnitude of the decrease in LPC concentrations could not be justified solely by the action of *B. divergens* in infected RBCs, given the parasitemias used in this study (≈40%), as LPCs are likely to be constitutive of uninfected RBCs [39]. Consequently, these results indicate that LPC depletion occurs in both infected and non-infected RBCs coexisting in iRBC samples, pointing to a potential *B. divergens*-induced phospholipase activity in infected and uninfected host cells. This phospholipase activity is likely to provide LPC breakdown products, ultimately culminating in intracellular phospholipid biosynthesis needed for membrane biogenesis, a process that has been described in *P. falciparum* [40,41,42]. Interestingly, significantly increased levels of phosphocholine were observed in iRBCs (Table S1), further suggesting that LPCs are a relevant carbon and phosphate source for the synthesis of phospholipids in *B. divergens*. In addition to LPC depletion, a decrease in a C18:1 fatty acid (vaccenic acid) in iS may be related to *B. divergens* metabolism, as defined levels of a combination of fatty acids have been reported as essential for *P. falciparum* [43]. To the best of our knowledge, the potential role of vaccenic acid in *B. divergens* and other intraerythrocytic apicomplexa remains to be further elucidated.

### 3.3. Relationship Between Oxidative Stress and B. divergens Metabolism

Oxidative stress is closely linked to cellular redox status, which is primarily influenced by the generation of reactive oxygen and nitrogen species (ROS and RNS, respectively) and their detoxification through antioxidant mechanisms. Within RBCs, the intracellular environment is oxygen-rich, and oxidative stress is primarily counteracted by glutathione and its recycling via NADPH produced through the pentose phosphate pathway [44]. In the case of intraerythrocytic apicomplexans, increased oxidative stress has been reported in *Plasmodium* spp.-infected RBCs, and these parasites are known to be sensitive to the presence of ROS [45,46,47]. Interestingly, glucose-6-phosphate dehydrogenase deficiency has been widely associated with resistance to malaria [48], although the mechanisms underlying this protective effect remain poorly understood [49,50]. Both the host RBC and *B. divergens* pentose phosphate pathways likely play a relevant role in the host–parasite interaction, as suggested by the notable alterations in pentose phosphate compounds found in this study, pointing to disruptions in the redox status of iRBC samples (Figure 4 and Figure 5, Tables S1 and S2). We propose that targeting the pentose phosphate pathway could be explored as a therapeutic strategy in future studies, especially if the bifunctional glucose-6-phosphate dehydrogenase 6-phosphogluconate dehydrogenase is present, as this enzyme is structurally different from those composing the human pentose phosphate pathway, likely facilitating the development of selective inhibitors [51]. Further evidence on redox alterations in *B. divergens*-infected RBCs was supported by the selective presence of γ-glutamylcysteine in iRBCs (Table S1), decreased NAD+ levels observed in iRBCs, and concomitant increases in NADH (Figure 5, Table S2). Additional alterations in redox-related metabolites were also observed, including significant increases in S-glutathionylcysteine and cystine levels in iRBCs (Table S1). Further studies are required to elucidate the mechanisms underlying redox-related metabolic alterations during *B. divergens* infection and proliferation in RBCs.

### 3.4. Involvement of Metabolic Alterations in the Pathophysiology of Human Babesiosis

The depletion of major nutrients utilized by *B. divergens* may have important consequences for the infected host. In this study, we observed the relevant consumption of glucose, arginine, and glutamine occurring in the supernatants of iRBC cultures. These results suggest that human babesiosis may cause hypoglycemia, hypoargininemia, and hypoglutaminemia. This is supported by evidence that hypoglycemia has been associated with severe malaria cases [52,53], while hypoargininemia is a well-described feature of malaria patients and has been implicated in the disease pathogenesis by impairing erythrocyte deformability and reducing nitric oxide (NO) production [54,55]. Hypoglutaminemia, although less well understood, has been correlated with poor outcomes in critically ill patients [56]. Collectively, our findings suggest that depletion of these metabolites in plasma may result directly from the metabolic demands of *B. divergens*, as occurs in *P. falciparum* [35,57,58]. Given these findings, one might consider supplementing these metabolites to restore physiological levels in individuals. However, it remains unclear whether such supplementation would be beneficial to the host or might instead support parasite growth, resulting in poorer outcomes [59,60].

Regarding lipid metabolism, we observed a significant reduction in lysophosphatidylcholines (LPCs) in both iS and iRBCs. These results validate previous observations on LPC depletion in *B. divergens*-infected RBCs [28]. Notably, reduced plasma LPC levels have been reported in the plasma of malaria patients [61]. Despite the potential effects of LPC decrease in serum not being fully elucidated, LPC deprivation has been linked to gametocyte development in *P. falciparum* [62]. Therefore, it could be speculated that *B. divergens* gametocyte development could be coupled to glycerophospholipid availability.

Significant decreases in the abundances of extracellular metabolites were not limited to glucose, glutamine, arginine, and LPCs. Several additional compounds involved in biosynthetic and signaling pathways were also depleted, such as kynurenine, fumarate, glycerol, myo-inositol, 2-hydroxybutyric acid, 4-pyridoxic acid, and dihydroorotate. Altogether, these observations suggest that reduced levels of certain metabolites, which may affect relevant extracellular signaling pathways, may occur in the plasma of *B. divergens*-infected patients with high parasitemia. The functional consequences of these changes merit further investigation in the context of human babesiosis.

Despite the depletion of plasma compounds potentially contributing to the pathophysiology of *B. divergens* infection, the abnormal accumulation of certain compounds may also exert significant effects on the host. Among the metabolites detected in iSs but absent in uSs, free heme B emerged as the most prominent. Free heme is a well-established damage-associated molecular pattern (DAMP) inducing pro-inflammatory responses [63,64]. Notably, elevated levels of free heme have also been detected in untargeted metabolomic analyses of *P. falciparum*-infected RBC cultures [24], and several studies have highlighted its critical role in the pathogenesis of malaria [65]. Our findings suggest that *B. divergens* causes the release of free heme to the plasma of *B. divergens*-infected patients, which could have important implications for the host’s immune response. The origin of this release remains uncertain and may result from the interaction of the parasite with hemoglobin, an effect of net RBC damage, or the combination of both.

Another well-documented feature of malaria pathophysiology is the development of metabolic acidosis [10,25]. In *Plasmodium* infections, metabolic acidosis has been primarily attributed to anaerobic lactate production in host tissues as a consequence of parasite-induced hypoxia [25]. Studies have continued to characterize the biochemical components contributing to this phenomenon in malaria patients [25]. In the context of *B. divergens* infection and given the relevance of glucose and the TCA cycle in its metabolic activity, it is plausible that parasite-derived production and the extracellular release of organic acids may also contribute to acidosis in human babesiosis. In this study, we observed not only elevated levels of lactate in iS but also marked increases in pyruvate, both of which are likely major contributors to net organic acid production by *B. divergens*. Additionally, several other acidic metabolites were significantly increased in iS, including α-ketoglutarate, succinate, glutamate, nicotinate, orotate, phenylpyruvate, and 4-hydroxyphenylpyruvate (Figure S3, Table S1), which may serve as minor contributors to *B. divergens*-induced metabolic acidosis.

Beyond organic acids, the increased levels of sphinganines and sphingosines found in iRBCs and iS were consistent with previously described observations in iRBCs [28]. These sphingolipids are known to play roles in inflammatory signaling and may serve as triggers for host immune responses during infection [66]. Finally, a wide range of other metabolites showed increased abundances in iS (Table S1), including ADP, UMP, cytidine, thymidine, 2-deoxyAMP, 2-deoxycytidine, nicotinamide mononucleotide, 2-hydroxyglutarate, purine and pyrimidine-related compounds, and pentose phosphates. These compounds are typically intracellular, with many of them reflecting an ongoing parasite proliferation as they are produced in nucleotide metabolism during nucleic acid biosynthesis [28]. Their unexpected presence in the extracellular environment may trigger unknown host responses to *B. divergens* infection that are worthy of further investigation.

### 3.5. Uncharacterized Phosphorylation of HEPES in B. divergens-Infected RBCs

HEPES is a zwitterionic buffer widely used in cell culture media, such as RPMI 1640, with a pKa of 7.48 at 25 °C, which closely matches the physiological pH of human blood (~7.4). Although HEPES is primarily used for its buffering capacity, it contains a sulfonic acid group and has been detected in uRBC extracts, suggesting it may be taken up or retained by RBCs. In the present study, a phosphorylated derivative of HEPES (PEPES) was detected, displaying an additional phosphate moiety that may confer distinct buffering properties or chemical reactivity. To the best of our knowledge, PEPES has not been previously described in any chemical database or reported as a product of *B. divergens* metabolism in vitro. Under oxidative conditions, HEPES is known to generate reactive oxygen species (ROS), which can exert deleterious effects on cultured cells and potentially on intraerythrocytic parasites such as *B. divergens* [67,68]. However, the enzymatic or non-enzymatic mechanisms responsible for HEPES phosphorylation remain unknown. Further studies are required to confirm whether PEPES formation is biologically driven by *B. divergens* and whether it has any functional consequences for parasite growth under in vitro conditions.

## 4. Materials and Methods

### 4.1. Multiplatform Metabolomics Pipeline Structure

The present study followed a structure wherein untargeted multiplatform metabolomics was first used to analyze samples obtained from iRBCs, uRBCs, and supernatants from iRBC (iS) and uRBC (uS) cultures. Additionally, targeted metabolomics of polar compounds was performed in iRBCs, uRBCs, iS, and uS samples. The obtained metabolomics results were combined with the literature metabolic data of phylogenetically related organisms (Figure 2). Following isolation and metabolite extraction, individual sequence worklists were prepared for each type of sample (RBC or supernatant extract). Platform-specific metabolomics sample preparation, analysis, and data processing were carried out for GC-QTOF/MS, CE-TOF/MS, LC-QTOF/MS, and LC-QqQ/MS, as described below. After statistical analysis, high-level data integration using enrichment and pathway analyses was performed to provide a functional interpretation of the metabolomics results.

### 4.2. Reagents and Solutions

Methanol (Thermo Fisher Scientific, Loughborough, UK) was used as intracellular extraction solvent and as extracellular extraction solvent B. Acetonitrile (ACN, Thermo Fisher Scientific, Loughborough, UK) was used as extracellular extraction solvent A. All the solvents and reagents used in this study were of MS-grade quality.

The CE-TOF/MS sample solution was prepared by dissolving methionine sulfone (internal standard, Sigma-Aldrich, Steinheim, Germany) in Milli-Q water (Merck KGaA, Darmstadt, Germany) containing 0.1 M formic acid (Sigma-Aldrich, Steinheim, Germany) up to a 0.2 mM concentration. The CE-TOF/MS sheath liquid solution was prepared by mixing 100 mL of Milli-Q water with 100 mL of 100% methanol, 4 µL of concentrated formic acid, 10 µL of 5 mM purine, and 10 µL of 2.5 mM hexakis (1H,1H,3H-tetrafluoropropoxy)phosphazene HP722 (CE-TOF/MS reference masses, Agilent Technologies, Santa Clara, CA, USA). A CE-TOF/MS background electrolyte solution was prepared, consisting of 1M formic acid in methanol/water 1:9 (*v*/*v*).

LC-QTOF/MS mobile phase eluent A was 0.1% (*v*/*v*) formic acid in Milli-Q water. LC-QTOF/MS mobile phase eluent B was 0.1% (*v*/*v*) formic acid in ACN (Thermo Fisher Scientific, Loughborough, UK). The reference mass solution was 5% (*v*/*v*) Milli-Q water in ACN, containing three reference masses (hypoxanthine, ammonium trifluoroacetate, and (1H,1H,3H-tetrafluoropropoxy)phosphazene HP-0921) to enable online *m*/*z* correction and high mass resolution in the MS.

The GC-QTOF/MS ethoxymation solution was anhydrous pyridine containing 19 mg·mL^−1^ O-ethoxyamine (Sigma-Aldrich, Steinheim, Germany). The silylation solution was *N*-methyl-*N*-(trimethylsilyl)trifluoroacetamide (MSTFA; Sigma-Aldrich, Steinheim, Germany), with 1% trimethylchlorosilane (TCMS; Sigma-Aldrich, Steinheim, Germany). The GC/MS fatty acid methyl ester mix solution was prepared by 1:100 dilution of grain fatty acid methyl esters mix (C8:0-22:1, Sigma-Aldrich, Carlsbad, CA, USA) with dichloromethane (Carlo Erba Reagents, Sabadell, Spain). The GC/MS n-alkane mix was prepared by 1:5 dilution of C8-C40 Alkanes Calibration Standard with dichloromethane. The GC/MS internal standard solution was 10 mg·L^−1^ meso-erythritol (internal standard; Sigma-Aldrich, Steinheim, Germany) in Milli-Q water.

Different solutions were prepared for the LC-QqQ/MS determination of polar compounds. Mobile phase 1 eluent A was prepared by mixing 97% Milli-Q water (*v*/*v*) with 3% (*v*/*v*) liquid chromatography–mass spectrometry grade methanol (Thermo Fisher Scientific, Loughborough, UK), 10 mM tributylamine (≥99.5%) (Sigma-Aldrich, Steinheim, Germany), and 15 mM glacial acetic acid (VWR International, Fontenay-sous-Bois, France). Mobile phase 1 eluent B was prepared by adding 10 mM tributylamine and 15 mM glacial acetic acid to liquid chromatography–mass spectrometry grade methanol (Sigma-Aldrich, Steinheim, Germany). Mobile phase 2 eluent C was identical to mobile phase 1 eluent A. LC-QqQ/MS mobile phase 2 eluent D was ACN (Sigma-Aldrich, Steinheim, Germany). A solution of 50.09 µM p-chlorophenylalanine (Sigma-Aldrich, Steinheim, Germany) in Milli-Q water was used as the LC-QqQ/MS internal standard solution. A standard solution was prepared by mixing stock solutions of adenosine-5′-monophosphate, adenosine-5′-diphosphate, alpha-ketoglutarate, citric acid trisodium salt, D-fructose-6-phosphate disodium salt hydrate, fumaric acid, D-glucose-6-phosphate sodium salt, D-ribose-5-phosphate disodium salt hydrate, D-ribulose-5-phosphate sodium salt, D-sedoheptulose-7-phosphate lithium salt, D-xylulose-5-phosphate lithium salt, DL-isocitric acid trisodium salt hydrate, dihydroxyacetone phosphate lithium salt, L-glutamic acid, L-glutamine, L-arginine, L-asparagine, L-aspartic acid, L-citrulline, L-glutathione oxidized, L-histidine, L-isoleucine, L-lactic acid, L-leucine, L-malic acid, L-methionine, L-tryptophan, L-tyrosine, L-phenylalanine, L-serine, L-threonine, phosphoenolpyruvic acid sodium salt, pyruvic acid, succinic acid, uracil, β-nicotinamide adenine dinucleotide hydrate, β-nicotinamide adenine dinucleotide reduced disodium salt hydrate (all from Sigma-Aldrich, Steinheim, Germany), L-proline, and itaconic acid (both from Fluka, Buchs, Switzerland). Next, 1:2, 1:4, 1:8, 1:32, 1:64, 1:128, and 1:512 dilution series were generated from this standard solution by adding Milli-Q water.

Eluents composing the mobile phase for LC-QqQ/MS analysis of 2-(4-(2-(phosphonooxy)ethyl)piperazinyl)-ethanesulfonic acid were eluent A, which consisted of 10 mM ammonium acetate and 2.5 μM medronic acid (deactivator; Agilent Technologies, Santa Clara, CA, USA) in Milli-Q water, and eluent B, composed of 10 mM ammonium acetate and 2.5 μM medronic acid in water/acetonitrile (15:75, *v*/*v*).

### 4.3. Babesia divergens Strains and Erythrocyte Infection

#### 4.3.1. Ethics Statement

Human A+ blood from healthy volunteer donors was utilized to maintain blood-stage cultures of *B. divergens*. Donors provided written informed consent for the use of their blood for research purposes. Blood and the protocols for its use were approved for use by the Blood Transfusion Center in Madrid, Spain. Methods were performed according to the relevant guidelines and regulations of the institution.

#### 4.3.2. Parasite Propagation

In vitro cultures of *B. divergens* asynchronous cultures (Bd Rouen 1987 strain) were maintained, as previously described [69]. Briefly, the culture consists of a mixture of human A+ erythrocytes at 5% hematocrit in RPMI 1640 (Gibco, Grand Island, NY, USA) supplemented with 10% human serum (The Interstate Companies, Memphis, TN, USA), 7.5% (*w*/*v*) sodium bicarbonate solution (Lonza Group Ltd., Basel, Switzerland), and 50 μg/mL hypoxanthine (Sigma-Aldrich, Steinheim, Germany). Culture flasks containing non-infected RBCs (negative controls) were resuspended in the same medium as *B. divergens*-infected RBC cultures and maintained in vitro under the same conditions. Cells were cultured at 37 °C in a humidified atmosphere of 5% CO_2_.

### 4.4. Isolation of Intraerythrocytic Parasites and Supernatants from B. divergens In Vitro Cultures

*Babesia divergens* intraerythrocytic parasites and supernatants were collected from highly parasitized (≈40% of parasitemia) independent asynchronous *B. divergens* flasks. To collect *B. divergens* intraerythrocytic parasites, culture flasks were centrifuged (600× *g*, T = 4 °C, t = 5 min) in fixed-angle rotor heads to collect pellets (iRBCs). Then, the resulting pellets, containing a mix of *B. divergens* iRBCs and uRBCs, were washed and subsequently centrifuged three times (2000× *g*, T = 4 °C, t = 5 min), resuspended in RPMI, and placed on ice. Supernatants (iS) were collected from infected *B. divergens* cultures by two consecutive rounds of centrifugation at (2000× *g* and 8000× *g*, T = 4 °C, t = 5 min), respectively, and stored at −80 °C for further processing. Non-infected RBC pellets (uRBCs) and supernatants from non-infected cultures (uS) were isolated following the same protocol described above. Detailed information on biological replicates used in each analytical platform can be found in Table S5.

### 4.5. Metabolite Extraction

A multi-step extraction procedure was followed to quench metabolism, produce cell disruption, and enhance metabolite recovery, according to an adapted protocol [70]. Briefly, 800 μL of cold (−20 °C) intracellular extraction solvent was added to 200 μL of each iRBC and uRBC. Then, two subsequent freeze–thaw cycles (freezing in liquid nitrogen for 10 min, followed by thawing in an ice bath and vortexing gently) were performed, and after clearing by centrifugation (5725× *g*, T = 4 °C, t = 5 min), the metabolite extracts were transferred to independent vials. The remaining pellet was re-extracted twice, with sequential additions of 400 μL of cold (−20 °C) intracellular extraction solvent, followed by two freeze–thaw cycles and centrifugation under the above-described conditions. All supernatants containing intracellular extracts from the same sample were combined and stored at −80 °C for further sample processing and analysis. For untargeted CE-TOF/MS, GC-QTOF/MS, and LC-QTOF/MS analyses, iS and uS samples were quenched and extracted with cold (−20 °C) extracellular extraction solvent A in a 1:4 proportion (*v*/*v*). For targeted LC-QqQ/MS analyses, extracellular extraction solvent B was used in a 1:3 proportion (*v*/*v*). After metabolic quenching and extraction, supernatant samples were filtered through 0.22 μm MS^®^ Nylon syringe filters (Membrane Solutions, Plano, TX, USA) and stored at −80 °C for further sample processing and analysis.

### 4.6. CE-TOF/MS Analyses

Samples were prepared and analyzed using an adapted protocol described previously for CE/MS analysis [71]. Briefly, 250 μL of iRBC, uRBC, iS, and uS extracts were evaporated to dryness under high vacuum. The dried samples were resuspended in 50 μL of the sample solution by vortexing for 1 min. After subsequent centrifugation (12,600× *g*, T = 4 °C, t = 15 min), the resulting clear solution was analyzed by CE-TOF/MS using a CE System (Agilent 7100; Agilent Technologies, Santa Clara, CA, USA) coupled to a TOF/MS system (Agilent 6224; Agilent Technologies, Santa Clara, CA, USA). The separation occurred in a fused-silica capillary (96 cm total length, 50 μm i.d.; Agilent Technologies, Santa Clara, CA, USA) under normal polarity with a background electrolyte containing 1.0 M formic acid in 10% (*v*/*v*) methanol at 20 °C. The sheath liquid flow was set to 6 µL·min^−1^. Samples were hydrodynamically injected at 50 mbar for 50 s and stacked by injecting background electrolyte at 100 mbar for 20 s. Then, samples were separated using a capillary voltage of 30 kV, achieving a current value of ~2.2 μA. Analysis of samples was performed under positive electrospray ionization (ESI) mode. The optimized MS parameters were fragmentor = 125 V, Skimmer = 65 V, octopole = 750 V, nebulizer pressure = 10 psi, drying gas temperature = 200 °C, and drying gas flow rate = 10.0 L·min^−1^. The capillary voltage was 3500 V. Data were acquired in full MS scan mode using a mass range from *m*/*z* 74 to 1000 and an acquisition rate of 1 spectrum·s^−1^. Reference mass correction was performed using *m*/*z* 121.0509 and 922.0098. Samples were analyzed in a randomized order. Pooled QC samples of intra- and extracellular extracts were obtained by mixing aliquots of the study samples. These QC samples were used for system equilibration and to monitor analytical stability along the batch.

For iRBC and uRBC samples, the resulting CE-TOF/MS data files were cleaned of background noise, and unrelated ions and compounds were integrated using the Batch Recursive Feature Extraction tool with Agilent MassHunter Profinder version B.06.00 software. After data filtering, molecular features were putatively annotated by comparison of their migration time and spectra with an in-house library of pure standards and the CEU Mass Mediator annotation tool [72,73]. A targeted search of metabolites detected in RBC extracts was performed in the supernatant data files using the Batch Targeted Feature Extraction tool in Agilent MassHunter Profinder version B.06.00.

### 4.7. GC-QTOF/MS Analyses

Samples were prepared and analyzed using an adapted protocol described previously for GC/MS analysis [74]. Briefly, 10 µL of ethoxymation solution and 12 µL of GC/MS internal standard solution were added to 240 μL of iRBC, uRBC, and supernatant samples. Then, the samples were evaporated to dryness under high vacuum. The obtained dried extracts were derivatized by an MPS autosampler for GC/MS analysis. Briefly, aldehyde and keto groups were first converted to O-ethyloximes by reaction with an additional 18 µL of 19 mg·mL^−1^ O-ethoxyamine in pyridine for 90 min at 40 °C. In a second step, acid hydrogen-containing metabolites were trimethylsilylated by reaction with 42 µL of silylation solution for 50 min at 40 °C to enhance the GC/MS metabolite coverage.

The analysis was performed on an Agilent Technologies 7890B GC system equipped with a Gerstel MPS autosampler and an Agilent Technologies 7200 accurate mass Q/TOF analyzer equipped with a chemical ionization (CI) source (both from Agilent Technologies, Santa Clara, CA, USA). First, 1 µL of sample was injected into a multimode inlet in splitless mode. The injector port was programmed to be held for 0.2 min at 150 °C and programmed at an increase rate of 870 °C·min^−1^ until reaching 320 °C. The liner (Agilent 5190-2293: 900 μL; Agilent Technologies, Santa Clara, CA, USA) was connected to a capillary column (Optima 726 analytical column, 60 m × 0.25 mm, 0.25 µm film thickness, 100% dimethylpolysiloxane stationary phase; Macherey-Nagel, Düren, Germany). Helium was used as the carrier gas at a flow rate of 1.3 mL·min^−1^. The column temperature was 80 °C for 1 min and then programmed to increase at a rate of 20 °C·min^−1^ until 200 °C. Then, the temperature was increased at a rate of 5 °C·min^−1^ until 235 °C, increased at a rate of 3 °C·min^−1^ until 270 °C, and finally increased at a rate of 20 °C·min^−1^ until 320 °C, which was maintained for 1 min. The total runtime was 29 min. Methane (20% of gas pressure) was used in a CI source operating at 150 °C in positive mode. Full MS scan mode was selected as the acquisition mode, operating in extended dynamic range (2 GHz), with a mass range from 70 to 900 *m*/*z* and an acquisition time of 3.33 spectra·s^−1^. Samples were analyzed in a randomized order. Pooled QC samples of intra- and extracellular extracts were obtained by mixing aliquots of the study samples. These QC samples were used for system equilibration and to monitor analytical stability along the batch.

The individual analytical fingerprints obtained from iRBC and uRBC data files were deconvoluted using MassHunter Unknown Analysis version B.07.00. Selected accurate mass ions were used for feature signal integration in Agilent MassHunter Quantitative Analysis for TOF B.07.00. To perform compound annotation, blank-substracted spectra from each quantifier ion used for compound integration were exported using Agilent MassHunter Workstation Software Qualitative Analysis B.06.00. Then, using Python (version 3.6.0) and the openpyxl package, exported files were screened for the presence of proton adducts, considering the exact mass differences between [M+H]^+^, [M-CH_3_]^+^, [M+C_2_H_5_]^+^, and [M+C_3_H_5_]^+^ ions. Correlation of the signals between the integrated *m*/*z* and the [M+H]^+^ ion was manually verified using Workstation Software Qualitative Analysis version B.06.00. Once [M+H]^+^ ions were verified, these were screened against an exact mass in-house positive chemical ionization library of metabolites (1054 entries) based on the FiehnLib library [75]. This in-house library contained calculated exact masses of ethoxymated and silylated metabolites and associated Fiehn retention indexes of compounds. Experimental Fiehn retention indexes were estimated for unknown compounds using the linear correlation between the retention times of fatty acid methyl esters obtained from the analysis of the GC/MS fatty acid methyl ester mix solution and its associated Fiehn retention indexes. Both accurate mass error and the Fiehn retention index were used as constraints for metabolite annotation. Unidentified molecular ions were subjected to a recursive exact mass search in the NIST library [76], where the n-alkane retention index was considered for metabolite annotation. A target search of metabolites detected in RBC extracts was performed in infected and uninfected supernatants using Agilent MassHunter Quantitative Analysis for TOF version B.07.00.

### 4.8. LC-QTOF/MS Analyses

Samples were prepared and analyzed using an adapted protocol described previously for LC/MS analysis [77]. Briefly, 100 μL of the uRBC, iRBC, and supernatant extracts were aliquoted into LC/MS vials after vortexing gently. A HPLC system (1200 series, Agilent Technologies, Waldbronn, Germany), equipped with a degasser, two binary pumps, and a thermostated autosampler coupled to an Agilent 6520 QTOF/MS system (Agilent Technologies, Waldbronn, Germany), was used in both positive and negative ESI polarity modes to broaden the metabolome coverage. For sample analysis, 10 μL of extract was injected into a thermostated (40 °C) Discovery HS C18 column (150 × 2.1 mm, 3 μm; Supelco, Bellefonte, PA, USA) with a Discovery HS C18 guard column (20 × 2.1 mm, 3 μm; Supelco, Bellefonte, PA, USA). The mobile phase flow rate was 0.6 mL·min^−1^ for analysis in both positive and negative ionization modes. Initial conditions at time 0 were 25% B, increasing to 95% B in 35 min. The conditions were then returned to the starting conditions by 36 min, followed by a 9 min re-equilibration time. The total run time of the method was 45 min. Capillary voltage was set at 3.5 kV; the drying gas flow rate was 10.5 L·min^−1^ at 330 °C; the gas nebulizer was set to 52 psi; fragmentor voltage, skimmer voltage, and octopole radio frequency voltage were set to 175, 65, and 750 V, respectively. Mass spectrometry detection was performed in both positive and negative ESI polarity modes in full MS scan acquisition mode with an acquisition range from 50 to 1100 *m*/*z* and an acquisition rate of 1.2 spectra·s^−1^. Mass correction was performed using *m*/*z* 121.0509 and 922.0098 mass calibrators in the positive ESI polarity mode. *m*/*z* 112.9856, 966.0007, and 119.0363 were used for accurate mass correction in the negative ESI polarity mode. Samples were analyzed in separate runs (positive and negative ionization modes) in a randomized order. Pooled QC samples of intra- and extracellular extracts were obtained by mixing aliquots of the study samples. These QC samples were used for system equilibration and to monitor analytical stability along the batch. Given the similar number and identity of significant metabolite alterations found in the endometabolome by LC-ESI(+)-QTOF/MS and LC-ESI(-)-QTOF/MS, analysis of iSs and uSs was only performed by LC-ESI(+)-QTOF/MS.

For iRBC and uRBC samples, the resulting LC-QTOF/MS data files were cleaned of background noise and unrelated ions, and compound integration was performed using the Batch Recursive Feature Extraction tool using Agilent MassHunter Profinder version B.06.00. After data filtering, molecular features were putatively annotated by comparison of their retention time and spectra with an in-house library of pure standards and the CEU Mass Mediator tool [72]. A targeted search for metabolites found in RBC extracts was performed in the supernatant data files using the Batch Targeted Feature Extraction tool in Agilent MassHunter Profinder version B.06.00. After determination of statistically significant metabolites, an LC-QTOF/MS/MS analysis under positive polarity targeting relevant features was performed under the above-described chromatographic conditions. Tandem mass spectra were collected from features determined as relevant by targeting their *m*/*z* values as precursor ions (isolation width ≈ 1.3 Da), which were subjected to collision-induced dissociation using a ramped collision energy with slope and offset values of 3.8 and 4.6, respectively. MS/MS spectra were compared to experimental spectra collections present in the METLIN database [78] and in silico spectra using MetFrag [79].

### 4.9. LC-QqQ/MS Profiling of Polar Metabolites

First, 12 µL of LC-QqQ/MS internal standard solution was added to 300 μL of each methanolic (from uRBCs and iRBCs) and MeOH:H_2_O (3:1, *v*/*v*, from iSs and uSs) extract. Then, samples were evaporated under high vacuum. Subsequently, samples were resuspended in 60 μL of Milli-Q water with the use of a sonicator bath for 5 min. Vials were centrifuged at (3000× *g*, T = 4 °C, t = 10 min) prior to injection.

The LC/MS/MS analyses were performed in an Agilent 1290 Infinity (Agilent Technologies, Waldbronn, Germany) using a 1290 Infinity Binary Pump (1200 bar) and a 1260 Infinity Quaternary Pump (400 bar). The LC system was coupled to an Agilent 6460 (Agilent Technologies, Waldbronn, Germany) triple quadrupole mass spectrometer using an ESI interface working in multiple reaction monitoring (MRM) mode. The chromatographic method, the MS parameters, and the setup arrangement were based on a previously described method, with minor modifications [80]. Two MS methods under the same conditions were run: one aimed at quantifying specific metabolites (Table S2) and the other for complementary semiquantitative analysis covering other metabolites than the former (Table S2). The transitions showing the highest signal-to-noise ratios were used for the quantification of the analytes in samples (Table S1, LC-QqQ/MS data). Samples were analyzed in a randomized order. The output raw data files were preprocessed with Agilent MassHunter Workstation Software Qualitative Analysis for QQQ version B.08.00, from which metabolite matrices containing the integrated areas and RT for specific compounds were obtained and subjected to blank subtraction. Additionally, the absolute concentrations of a subset of metabolites were calculated for iRBCs and uRBCs (Table S2).

### 4.10. LC-QqQ/MS Targeted Analysis of 2-(4-(2-(Phosphonooxy)ethyl)piperazinyl)-ethanesulfonic Acid (PEPES)

First, 300 μL of a pooled sample obtained by mixing equal volumes of additional biological replicates of iRBC extracts (*n* = 3) was evaporated under high vacuum. Then, the sample was resuspended in 50 μL of acetonitrile using a sonicator bath for 5 min. Vials were centrifuged (3000× *g*, T = 4 °C, t = 10 min) prior to injection.

The LC/MS/MS analysis was performed in an Agilent 1290 Infinity HPLC system (Agilent Technologies, Waldbronn, Germany) coupled to an Agilent 6470 triple quadrupole (Agilent Technologies, Waldbronn, Germany) mass spectrometer equipped with an ESI source. Next, 10 μL of a resuspended iRBC sample pool was injected into a thermostated (50 °C) Agilent InfinityLab Poroshell 120 HILIC-Z, P column (2.1 mm × 150 mm, 2.7 μm; Agilent Technologies, CA, USA), equipped with an Agilent InfinityLab Poroshell 120 HILIC-Z guard column (2.1 mm × 5 mm, 2.7 µm; Agilent Technologies, CA, USA). The chromatographic gradient was programmed as follows: first, 96% B was maintained until 2 min after the start of analysis. Then, the proportion of B was progressively decreased until reaching 88% at 5.50 min, which was held until 8.50 min. Subsequently, the proportion of B was decreased to 86% at 9 min, and this proportion was held until 14 min. Next, %B was decreased until reaching 82% at 17 min. Finally, %B was progressively decreased until reaching 35% at 23 min, a proportion held for one minute before returning to initial conditions (96% B), which were maintained for 13 min. During a total method runtime of 39 min, a constant mobile phase flow of 0.250 mL·min^−1^ was maintained. The ESI source operated in negative polarity. The sheath gas was supplied at 225 °C and a flow rate of 13 L·min^−1^. The capillary voltage was set to 3500 V. Fragmentor, collision energy, and cell accelerator voltages were set to 135 V, 20 V, and 5 V, respectively. The detection in the MS equipment was configured in product ion scan mode, where a single precursor corresponding to *m*/*z* 317 was isolated across the entire method runtime.

### 4.11. Parasite Growth Assays Using Extracellular Nutrients

All reagents and media used in the growth assays were purchased from Sigma-Aldrich (Steinheim, Germany). For the glucose supplementation experiments, RPMI-1640 Medium without glucose (catalog number R1383) was used as the basal medium. D-glucose was added at final concentrations of either 2 g/L (corresponding to the standard RPMI concentration) or 4 g/L (representing a high concentration). A customized base medium (R5886) formulated without L-glutamine was used for the glutamine supplementation experiments. L-glutamine was added at either 0.3 g/L (corresponding to the standard RPMI concentration) or 0.6 g/L (representing a high concentration). For hypoxanthine supplementation experiments, standard RPMI 1640 medium (catalog number R8758), containing all essential nutrients, was supplemented with 50 mg/L of hypoxanthine. The culture medium supplemented with D-glucose (2.0 g/L), L-glutamine (0.3 g/L), and L-arginine (0.2 g/L)—each added at their standard RPMI concentrations—served as the reference condition for the hypoxanthine supplementation experiments. All media formulations were supplemented with 10% human serum (blood type: A positive; Interstate Blood Bank Inc., Memphis, TN, USA), in accordance with the standard complete medium used throughout this study for the routine propagation of the parasites.

Free merozoites of *B. divergens* were isolated from in vitro cultures, as previously described [81]. Naïve human red blood cells (RBCs) at a haematocrit of 5% were infected with the free merozoites in triplicate 6-well culture plates, each containing one of the previously described media prepared with or without supplementation of glucose or L-glutamine. The cultures were incubated at 37 °C under 5% CO_2_, with daily medium changes. Parasite invasion and intracellular development were monitored using Giemsa-stained thin blood smears. For the glucose evaluation, samples were collected at 6, 12, 24, and 48 h post-infection. For the glutamine evaluation, samples were analyzed at 24 and 48 h post-infection. Parasitemia was quantified by manually counting infected RBCs in contiguous fields until a total of 2000 cells per sample had been evaluated.

For the hypoxanthine supplementation experiments, *B. divergens* cultures were initiated at approximately 0.5% parasitemia. Experiments were performed in triplicate using RPMI 1640 medium supplemented with 50 mg/L hypoxanthine, alongside control cultures in RPMI 1640 medium without hypoxanthine. Cultures were maintained in 6-well plates, with daily medium changes over a period of 3 days (equivalent to 72 h, one experiment) or 2 days (equivalent to 48 h, two confirmatory experiments).

Parasitemia growth was monitored by Giemsa-stained thin blood smears, and parasitemia was quantified by manually counting infected RBCs in contiguous fields until a total of 2000 cells per sample.

### 4.12. Statistical Analysis

Total Useful Signal (TUS) normalization was performed in CE-TOF/MS, GC-QTOF/MS, LC-QTOF(+)/MS, and LC-QTOF(-)/MS data matrices. Both LC-ESI-(-)-QqQ/MS data matrices were subjected to internal standard normalization, and specifically, the LC-ESI(-)-QqQ/MS data matrix was subjected to a second TUS normalization to provide data harmonization with untargeted data. Subsequently, the data matrices were adapted to a MetaboAnalyst (version 5.0) [82] format. Then, the metabolite matrices were uploaded to the MetaboAnalyst server, using the default MetaboAnalyst missing value imputation (LoD replacement, 1/5 of the minimum positive value of each variable). The statistical significance of metabolic features was determined using the t-test, followed by FDR (Benjamini-Hochberg) correction in two different pairwise comparisons (iRBCs/uRBCs and iSs/uSs) using MetaboAnalyst (v. 5.0). An adjusted *p*-value of 0.01 was considered as a cutoff for statistical significance. Multivariate principal component analysis (PCA) was generated for the pairwise comparisons mentioned above using SIMCA-P version 14.1 (Umetrics, Umeå, Sweden). Individual univariate statistical tests were performed for each metabolite matrix (GC-QTOF/MS, CE-TOF/MS, LC-ESI(+)-QTOF/MS, LC-ESI(-)-QTOF/MS, and LC-ESI(-)-QqQ/MS).

For statistical analysis of growth experiments, a two-way analysis of variance (ANOVA) was applied to evaluate the effects of metabolite concentration and time on growth (Tables S3 and S4). Initially, two-way ANOVA was conducted to assess the main effects of each factor (metabolite concentration and time) and their interaction using Python (version 3.11). This approach was applied independently to experiments performed under different supplementation conditions, including glucose, glutamine, and hypoxanthine. Post-hoc pairwise comparisons were performed using Tukey’s Honest Significant Difference (HSD) test with Python (version 3.11). This method applies a family-wise error rate adjustment to account for multiple testing and to control the overall Type I error (FWER-adjusted *p*-values using the Tukey–Kramer method).

### 4.13. Metabolic Pathway Analysis

Kyoto Encyclopedia of Genes and Genomes (KEGG) compound identifiers [83] were matched with annotated and identified metabolites. First, unique annotations were matched to their specific KEGG compound ID. When multiple possible stereoisomers were compatible with a metabolite annotation, the KEGG compound ID corresponding to the most expected metabolite in eukaryotic organisms was assigned. When possible, generic KEGG compound identifiers were assigned to metabolites without a unique KEGG compound ID representation in the KEGG database. Multiple KEGG compound identifiers were given to potential compounds with multiple annotations and considered individual metabolites for pathway analyses. To obtain a view of the representation of multiple pathways and metabolites present in the distinct types of samples obtained from intraerythrocytic parasites and their respective negative controls, chemical over-representation analysis (CORA) and over-representation analysis (ORA) utilizing the KEGG metabolite sets obtained from human pathways were performed using MetaboAnalyst (v. 5.0) [82]. For both CORA and ORA, the default settings were selected on the server. To identify the global compendium of metabolic pathways altered in the intracellular media of the cells composing the pellet of iRBCs and their supernatants, pathway analysis (PA) utilizing the Plasmodium 3D7 pathway set from KEGG was performed for KEGG compound lists obtained from significantly altered metabolites obtained from the comparisons of iRBCs with uRBCs and of iSs with uSs. Adjusted *p*-values obtained in ORA and PA were calculated using FDR correction over *p*-values obtained in hypergeometric tests (an FDR significance threshold of 0.05). Relative-betweenness centrality was selected as a parameter for topology analysis performed in PA. To further characterize and group the altered pathways and altered metabolite types, compounds present in pellet and supernatant extracts were classified according to their significance and fold-change sign in the two comparisons described above. Then, KEGG compound lists obtained from each group of metabolites were subjected to CORA and PA, using identical conditions, as described above.

## 5. Conclusions

The combination of distinct analytical platforms used in this study has provided a comprehensive overview of the metabolome related to *B. divergens* infection in vitro. Profound metabolic alterations occur upon *B. divergens* infection, arising from the parasite’s metabolic activity, as well as from the RBC-*B. divergens* system and the extracellular environment, rendering a unique metabolic status of *B. divergens* in vitro cultures. A wide panel of metabolites with differential concentrations between infected and uninfected RBCs was found. Although the sample size was a limitation of the study, metabolites showing altered levels could be further evaluated as diagnostic, prognostic, or treatment biomarkers in future in vivo studies using large cohorts. Of particular relevance are the compounds that were only detected in both infected RBCs and in the supernatant, including distinct pyrimidine and purine-related compounds, as these are not expected to be found in RBCs lacking nuclei.

The compendium of metabolic alterations found in the study pointed to unresolved aspects of *B. divergens* metabolism and cellular biology. In particular, the provided insights related to the major carbon and nitrogen sources, such as glucose, glutamine, arginine, and lysophosphatidylcholines, as well as the central carbon and redox metabolism of *B. divergens* helped us to highlight metabolic pathways whose inhibition may have potential therapeutic implications, affecting the growth of the intraerythrocytic form of the *B. divergens* intraerythrocytic parasite. Furthermore, we provide insights into the potential mechanisms underlying distinct aspects of *B. divergens* pathogenesis that are to be further confirmed in vivo, such as a potential hypoargininemia, hypoglutaminemia, hypoglycemia, and signaling effects mediated by increased intracellular metabolites in the extracellular medium.

Ultimately, this study may serve as a preliminary resource for future mechanistic and in vivo confirmatory studies. Integration of metabolomic data with other omic data, such as that obtained from genomic, transcriptomic, and proteomic analyses, could be further considered to provide novel insights into the mechanisms underlying the metabolite alterations described in this study.

As babesiosis remains a health threat, especially in immunocompromised individuals and blood transfusion recipients, these findings provide valuable insights that can contribute to the development of more effective interventions.

## Figures and Tables

**Table 1:** Chemical over-representation analysis (CORA) and the most enriched pathway term found in pathway analyses of significant metabolites present in iRBCs, iSs, uRBCs, and uSs, classified according to their significance and fold change. Metabolites were grouped according to the trend in mean values in iRBCs and iSs, denoted as [iRBC trend in means]/[iS trend in means]. In this context, “+” indicates a statistically significant increase in relative abundance; “-“ indicates a statistically significant decrease; “ns” denotes non-significant changes; * denotes compound with *p* < 0.05 and FDR < 0.05.

Group	CORA Enriched Metabolite Sets (*p* < 0.05)	Enriched Pathway Terms (*p* < 0.05)
+/+	amines; pyridoxines; alpha-keto acids and derivatives; phenylpyruvic acid derivatives; carboximidic acids; benzenediols; pyrimidines and pyrimidine derivatives; amino acids, peptides, and analogs	-
+/ns	amino acids, peptides and analogs; purine 2′-deoxyribonucleosides; phosphate esters; linoleic acid derivatives	-
ns/+	carbohydrates and carbohydrate conjugates *; purines and purine derivatives *; purine ribonucleotides *; pyrimidines and pyrimidine derivatives; pyrimidine ribonucleosides; glycosylamines; alpha-hydroxyacids and derivatives; purine ribonucleosides; amino acids, peptides and analogs; phenylpyruvic acid derivatives; pyrimidine deoxyribonucleotides; gamma-ketoacids and derivatives; pyrimidine ribonucleotides; beta-hydroxyacids and derivatives; phenylmethylamines; dicarboxylic acids and derivatives; phosphate esters; pyridinedicarboxylic acids and derivatives	carbon fixation by Calvin cycle *; pyrimidine metabolism; pentose and glucuronate interconversions; citrate cycle (TCA cycle); purine metabolism; pentose phosphate pathway
-/-	amino acids, peptides, and analogs; glycerophosphocolines	-
-/ns	Carbohydrates and carbohydrate conjugates *; amino acids, peptides, and analogs; benzoic acids and derivatives	-
ns/-	Carbohydrates and carbohydrate conjugates *; tricarboxylic acids and derivatives *; alpha-hydroxyacids and derivatives; sphingoid base analogs; dicarboxylic acids and derivatives; pyridinecarboxylic acids and derivatives; alcohols and polyols	glyoxylate and dicarboxylate metabolism *; citrate cycle (TCA cycle); alaninine, aspartate, and glutamate metabolism
+/-	Phosphosphingolipids; amines	-
-/+	Quaternary ammonium salts	-

## Data Availability

Mass spectrometry data was uploaded to the Metabolomics Workbench Repository (dataset ID: ST001878) (https://www.metabolomicsworkbench.org/data/DRCCMetadata.php?Mode=Study&StudyID=ST001878, accessed on 1 August 2025).

## Supplementary Materials

The following supporting information can be downloaded at: https://www.mdpi.com/article/10.3390/ijms26167677/s1.
