# Supplementary material for: Application of Multiplatform Mass Spectrometry to the Study of Babesia divergens Metabolism and the Pathogenesis of Human Babesiosis
Source: Int J Mol Sci. 2025 Aug 8;26(16):7677. doi: 10.3390/ijms26167677 (PMC12386598; doi:10.3390/ijms26167677)
Supplement: Supplementary file 1 [file ijms-26-07677-s001.zip › File S1.pdf]

File S1. Detailed information on the data and findings used for structural elucidation of 2-(4-(2-(phosphonooxy)ethyl)piperazinyl)-ethanesulfonic acid (PEPES).

Detailed evaluation of coeluting signals revealed highly-correlated *m/z* corresponding to $[M+Na]^+$ and $[M+K]^+$ adducts, confirming a molecular mass of 318.0654 Da (ppm error ≈ 10). The compound also produced an in-source fragmentation at *m/z* 221.0954 (ppm error ≈ 10), which could be attributed to a loss of water from the protonated adduct of 4-(2-hydroxyethyl)-1-piperazineethanesulfonic acid, a buffering agent added to the cell cultures (HEPES). The mass difference between the molecular masses of this unknown compound and HEPES corresponded to a difference of HPO_3 (m/z difference of 79.9639, ppm error ≈ 10), leading to the candidate formula C8H19N2O7PS, notable isotopic distribution scores were calculated ($[M+H]^+$ ion, first candidate, >99% score). Evaluating the structure of HEPES, the most logical candidate compatible with the in-source fragment was a phosphorylation of HEPES free -OH moiety, giving 2-(4-(2-(phosphonooxy)ethyl)piperazinyl)-ethanesulfonic acid (PEPES). To further validate this annotation, tandem mass-spectrometry by a complementary LC-QqQ/MS analysis of iRBC samples in negative ESI mode. Targeting of the precursor ion for the potential PEPES (*m/z* 317, [M-H]-), MS/MS spectra showed (i) a water loss from deprotonated PEPES, (ii) a peak corresponding to HEPES (m/z 237), (iii) a characteristic fragment representing a phosphate loss (*m/z* 219), and fragments corresponding to phosphate and metaphosphate ions (*m/z* 79 and *m/z* 97, respectively). These results collectively confirm the identity of the compound as PEPES corroborating that HEPES is selectively phosphorylated in *B. divergens* RBC cultures (Figures 6c and 6d).
