# Supplementary figures and images for: Application of Multiplatform Mass Spectrometry to the Study of Babesia divergens Metabolism and the Pathogenesis of Human Babesiosis
Source: Int J Mol Sci. 2025 Aug 8;26(16):7677. doi: 10.3390/ijms26167677 (PMC12386598; doi:10.3390/ijms26167677)

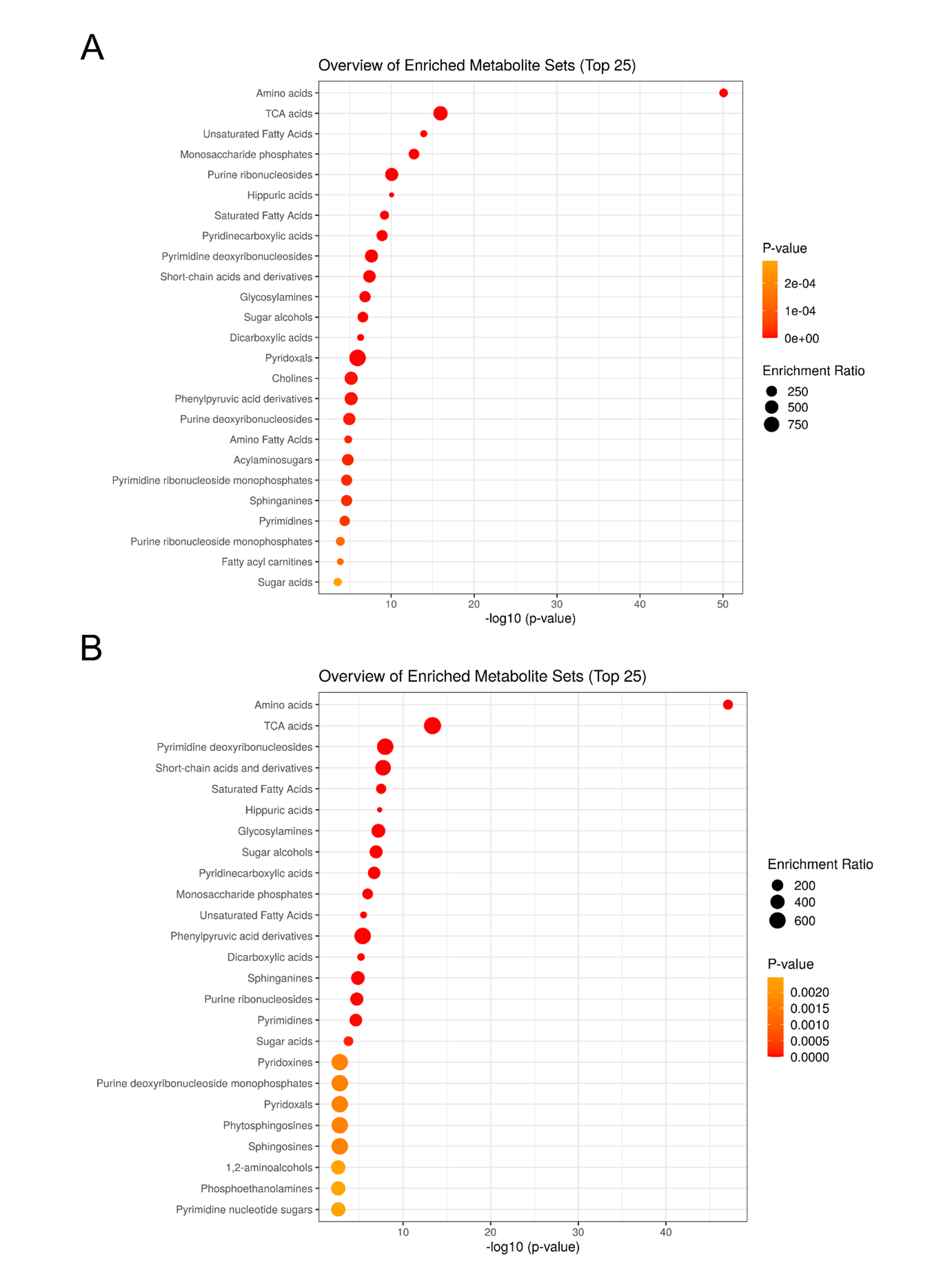

Supplement: Supplementary file 1 [file ijms-26-07677-s001.zip › Figure S1.png]

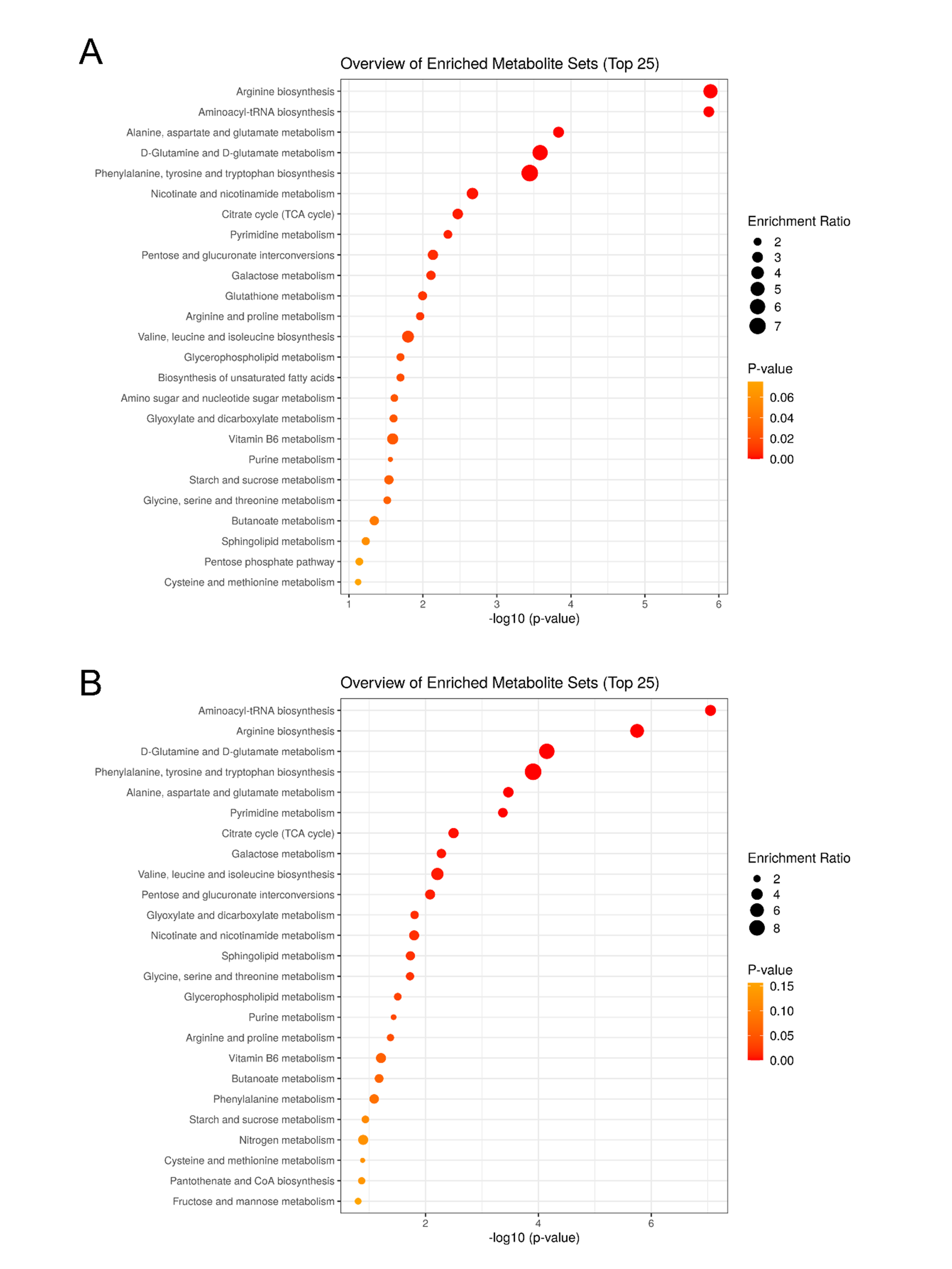

Supplement: Supplementary file 1 [file ijms-26-07677-s001.zip › Figure S2.png]

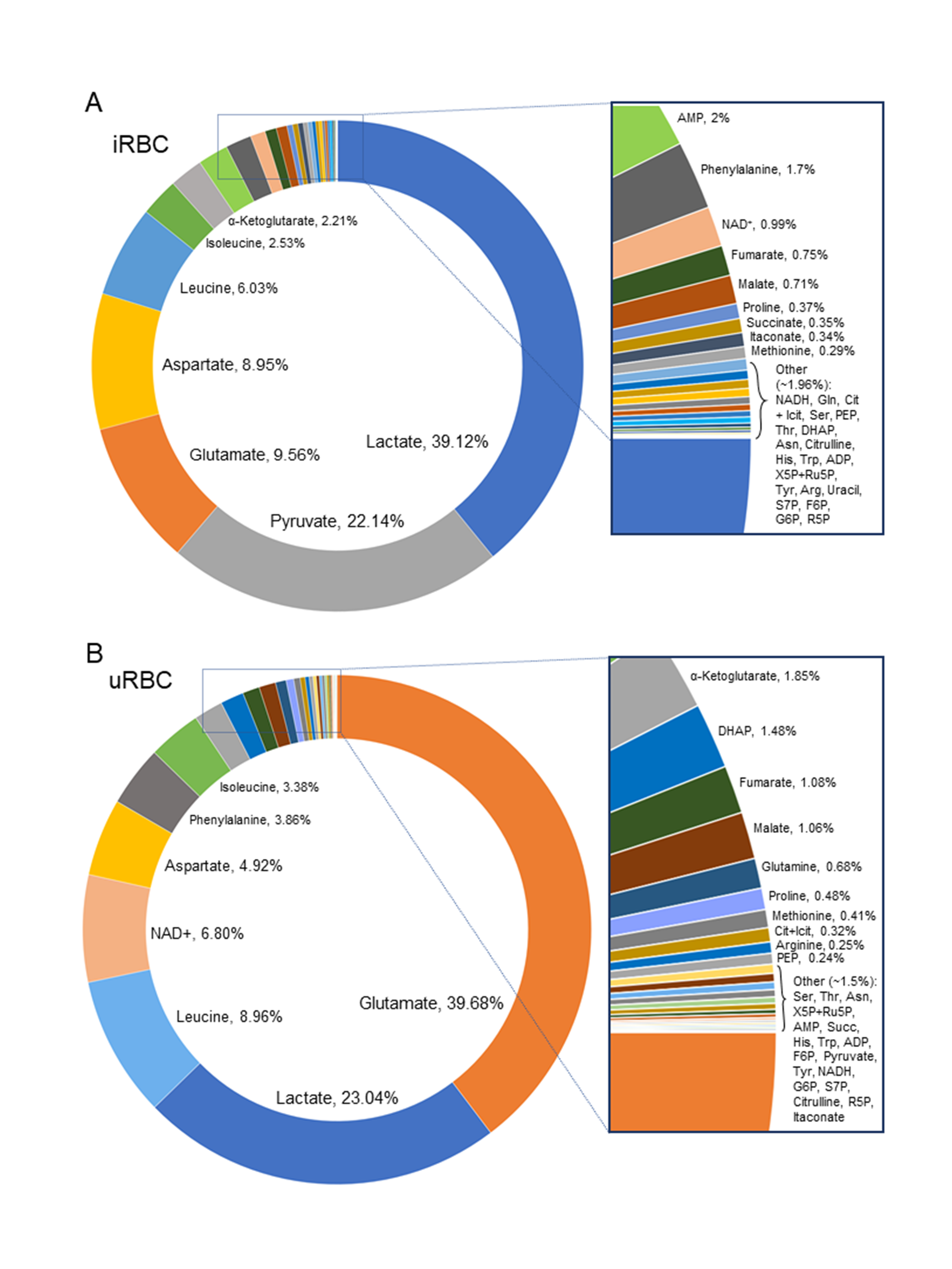

Supplement: Supplementary file 1 [file ijms-26-07677-s001.zip › Figure S3.png]
